# Neurotechnological Approaches to Cognitive Rehabilitation in Mild Cognitive Impairment: A Systematic Review of Neuromodulation, EEG, Virtual Reality, and Emerging AI Applications

**DOI:** 10.3390/brainsci15060582

**Published:** 2025-05-28

**Authors:** Evgenia Gkintoni, Stephanos P. Vassilopoulos, Georgios Nikolaou, Apostolos Vantarakis

**Affiliations:** 1 Department of Educational Sciences and Social Work, University of Patras, 26504 Patras, Greece; stephanosv@upatras.gr (S.P.V.); gnikolaou@upatras.gr (G.N.); 2 Lab of Public Health, Department of Medicine, University of Patras, 26504 Patras, Greece; avanta@upatras.gr

**Keywords:** mild cognitive impairment (MCI), cognitive rehabilitation, neuromodulation, electroencephalography (EEG), virtual reality (VR), cognitive training, artificial intelligence (AI), machine learning, neuroplasticity, digital biomarkers, personalized interventions, non-invasive brain stimulation, brain–computer interface, neurofeedback, physical exercise, multimodal interventions

## Abstract

*Background/Objectives:* Mild Cognitive Impairment (MCI) represents a clinical syndrome characterized by cognitive decline greater than expected for an individual’s age and education level but not severe enough to significantly interfere with daily activities, with variable trajectories that may remain stable, progress to dementia, or occasionally revert to normal cognition. This systematic review examines neurotechnological approaches to cognitive rehabilitation in MCI populations, including neuromodulation, electroencephalography (EEG), virtual reality (VR), cognitive training, physical exercise, and artificial intelligence (AI) applications. *Methods:* A systematic review following PRISMA guidelines was conducted on 34 empirical studies published between 2014 and 2024. Studies were identified through comprehensive database searches and included if they employed neurotechnological interventions targeting cognitive outcomes in individuals with MCI. *Results:* Evidence indicates promising outcomes across multiple intervention types. Neuromodulation techniques showed beneficial effects on memory and executive function. EEG analyses identified characteristic neurophysiological markers of MCI with potential for early detection and monitoring. Virtual reality enhanced assessment sensitivity and rehabilitation engagement through ecologically valid environments. Cognitive training demonstrated the most excellent efficacy with multi-domain, adaptive approaches. Physical exercise interventions yielded improvements through multiple neurobiological pathways. Emerging AI applications showed potential for personalized assessment and intervention through predictive modeling and adaptive algorithms. *Conclusions:* Neurotechnological approaches offer promising avenues for MCI rehabilitation, with the most substantial evidence for integrated interventions targeting multiple mechanisms. Neurophysiological monitoring provides valuable biomarkers for diagnosis and treatment response. Future research should focus on more extensive clinical trials, standardized protocols, and accessible implementation models to translate these technological advances into clinical practice.

## 1. Introduction

Mild Cognitive Impairment (MCI) is an intermediate stage between normal aging and dementia, marked by cognitive decline that exceeds typical age-related changes but does not yet interfere significantly with daily functioning [1,2,3]. Affecting approximately 15–20% of adults over 65, MCI is a growing public health concern and a key target for early intervention, especially given its 10–15% annual conversion rate to Alzheimer’s disease—substantially higher than the general elderly population [4,5,6,7].

MCI is a heterogeneous condition, classified into amnestic and non-amnestic subtypes, with single or multiple cognitive domains affected. This complexity poses challenges for both assessment and intervention. While traditional strategies such as pharmacotherapy, cognitive training, physical activity, and lifestyle changes have shown mixed results, technological innovations open new avenues for MCI management [8,9,10,11]. Recent developments include neuromodulation techniques like transcranial direct current stimulation (tDCS) and transcranial magnetic stimulation (TMS), which non-invasively target brain regions to enhance memory, attention, and executive function [12,13,14,15]. Electroencephalography (EEG) also plays a crucial role by capturing neurophysiological changes linked to MCI and aiding in biomarker discovery for early detection [16,17,18,19,20]. Meanwhile, virtual reality (VR) offers immersive, ecologically valid training environments that may improve engagement and facilitate real-world cognitive practice [21,22,23,24]. Physical exercise interventions—such as aerobic, resistance, and multimodal training—have demonstrated cognitive benefits through mechanisms like increased cerebral blood flow and neuroplasticity [25,26,27]. In parallel, artificial intelligence (AI) is beginning to influence MCI research by supporting early detection through digital biomarkers, predicting cognitive trajectories, and personalizing interventions using large-scale data analytics [28,29,30].

At the intersection of neuroscience and emerging technologies, these tools reshape how MCI is assessed, monitored, and treated. Enhanced real-time data analysis and adaptive interventions promise greater precision in addressing cognitive decline. This systematic review evaluates the current landscape of technological approaches in MCI rehabilitation, focusing on neuromodulation, EEG, VR, cognitive training, physical exercise, and AI. Our goal is to synthesize key findings and highlight directions for future research and clinical innovation [31,32,33].

## 2. Literature Review

### 2.1. Mild Cognitive Impairment (MCI): Pathophysiology and Neural Mechanisms

MCI is a heterogeneous syndrome involving cognitive decline greater than expected for age and education, yet without significant impairment in daily functioning. Neurophysiological research has identified several underlying mechanisms, including disrupted functional connectivity, altered neural oscillations, reduced synaptic plasticity, and increased neuroinflammation [34,35].

EEG studies have consistently shown early abnormalities in MCI, such as reduced alpha power, increased theta activity, and changes in event-related potentials (ERPs). These alterations—especially slowing of the posterior dominant rhythm, reduced signal complexity, and diminished inter-regional synchronization—may serve as early biomarkers of cognitive decline [36,37,38,39,40,41].

Functional MRI studies reveal altered activity in key networks, particularly the default mode network (DMN), with reduced resting-state connectivity and compensatory hyperactivation during cognitive tasks. Structural changes are also evident, notably reduced volume and activity in the medial temporal lobe, including the hippocampus and entorhinal cortex, and increased activation in the prefrontal cortex during memory tasks, suggesting compensatory mechanisms [42,43,44,45,46,47].

Diffusion tensor imaging (DTI) highlights disrupted white matter integrity in tracts such as the fornix, uncinate fasciculus, and corpus callosum, which are critical for memory and executive function. These structural and functional alterations provide a foundation for targeted interventions to preserve and enhance cognitive function in individuals with MCI [48,49,50,51,52,53,54].

### 2.2. Rehabilitation Approaches for MCI

Cognitive rehabilitation in MCI aims to enhance cognitive function and delay progression to dementia. Structured cognitive training programs targeting domains such as memory, attention, and executive function have shown modest but meaningful improvements. According to meta-analyses, multi-domain interventions tend to yield larger effects than single-domain approaches [55,56,57,58,59].

Strategy-based memory training, including mnemonic techniques like the method of loci, spaced retrieval, and visual imagery, has been particularly effective for amnestic MCI. While generalization to untrained tasks and daily functioning remains challenging, some studies report sustained cognitive gains for up to two years post-intervention [60,61,62].

Computer-based cognitive training has gained traction due to its adaptability and accessibility. These programs typically offer adaptive difficulty, real-time feedback, and engaging interfaces. Although results are promising, variability in design limits cross-study comparability [63,64,65,66].

Virtual reality (VR) has emerged as a powerful tool for assessment and intervention. VR environments offer realistic, controlled settings that closely simulate daily tasks. Studies show that VR-based memory training improves real-world functional outcomes better than traditional methods [67,68,69,70]. VR also supports dual-task training (combining cognitive and motor tasks), targets spatial navigation—a domain often impaired early in MCI—and enables remote intervention delivery, increasing accessibility for underserved populations [71,72,73,74,75,76,77].

Physical exercise is another evidence-based, non-pharmacological intervention for MCI. Aerobic training (3–5 sessions per week, 30–60 min) is associated with improved executive function, memory, and processing speed, likely mediated by increased cerebral blood flow, higher brain-derived neurotrophic factor (BDNF) levels, reduced inflammation, and enhanced neurogenesis [78,79,80]. Resistance training and mind-body practices like tai chi and yoga also show cognitive benefits, potentially through improved balance, reduced stress, and greater bodily awareness [81,82,83,84].

Multimodal approaches that combine cognitive training, physical exercise, and lifestyle modification are increasingly supported. Trials like the FINGER study demonstrate that targeting multiple risk factors can help preserve or enhance cognitive function in at-risk populations. Combined cognitive-physical training programs show synergistic benefits for both cognitive and physical health in MCI [85,86,87].

Telerehabilitation has gained momentum, especially since the COVID-19 pandemic, enabling the delivery of multimodal interventions via digital platforms. These approaches allow for structured, remotely monitored training, though challenges around digital literacy and access remain [88,89,90,91].

### 2.3. Neurophysiological and Neuroimaging Methodologies in MCI

EEG is a non-invasive, cost-effective method for assessing neurophysiological changes in MCI. Quantitative EEG (qEEG) metrics have shown strong potential in distinguishing MCI from normal aging and dementia, with reported sensitivity and specificity ranging from 75% to 85% [92,93,94]. Common EEG markers in MCI include increased theta power, reduced alpha power, lower signal complexity (e.g., entropy), and disrupted functional connectivity.

Event-related potentials (ERPs), particularly the P300 component, are consistently altered in MCI, with delayed latency and reduced amplitude linked to deficits in attention and working memory. Other ERP components like mismatch negativity (MMN) and N400 also show potential as early biomarkers for MCI detection and monitoring [95,96,97,98,99].

Advancements in EEG analysis—such as source localization, microstate segmentation, and graph theory—have enhanced its ability to characterize network-level dysfunction in MCI. These techniques help map disruptions in cognitive networks and may identify individuals at greater risk for progression to dementia [100,101,102,103,104].

Non-invasive brain stimulation methods, including tDCS and TMS, have emerged as promising therapeutic options. These approaches aim to boost neuroplasticity and modulate cortical activity in brain regions affected by MCI.

tDCS applied to the dorsolateral prefrontal cortex has been associated with improved working memory and executive function, with some effects persisting beyond the stimulation period [105,106,107,108,109,110]. Repetitive TMS (rTMS), especially high-frequency protocols targeting the prefrontal cortex, has shown benefits for memory and language. Combining neuromodulation with cognitive training may enhance and prolong intervention effects. Emerging personalized stimulation strategies—guided by individual EEG patterns—are being developed to optimize outcomes further [111,112,113,114,115].

### 2.4. AI Applications in MCI Management

Artificial intelligence (AI), particularly machine learning (ML), is transforming the early detection, diagnosis, and management of MCI. Deep learning models applied to structural neuroimaging data can detect subtle atrophy patterns predictive of MCI and Alzheimer’s disease risk, often outperforming conventional diagnostic methods. Convolutional neural networks (CNNs) have achieved 80–90% accuracy in classifying MCI versus healthy controls and in identifying MCI subtypes from MRI data [116,117,118].

ML techniques have also shown promise with EEG data. Algorithms such as support vector machines (SVMs), random forests, and deep neural networks can classify MCI using spectral, connectivity, and signal complexity features, achieving 75–85% accuracy [119,120,121,122]. These models uncover subtle patterns in neurophysiological activity that are difficult to detect through traditional analysis.

Multimodal approaches integrating EEG, MRI, genetic, and cognitive data enhance early detection. By combining complementary inputs, these models can identify MCI-related changes earlier and more accurately, offering opportunities for preemptive intervention [123,124,125].

AI is also advancing predictive modeling of MCI progression. Algorithms using longitudinal neuroimaging, cognitive assessments, and CSF biomarkers have achieved 80–90% accuracy in forecasting conversion to Alzheimer’s disease over 2–3 years [126,127]. Machine learning has also been applied to predict individual treatment responses, identifying baseline markers correlating with intervention outcomes. For example, reinforcement learning has been used to adapt cognitive training based on user performance, optimizing outcomes [128,129,130].

Natural language processing (NLP) adds another dimension by analyzing speech and language for early cognitive decline. Changes in lexical diversity, syntactic structure, and semantic coherence may serve as sensitive, non-invasive markers of MCI progression [131,132,133,134].

AI-driven cognitive interventions are emerging, though still in early development. Adaptive training platforms use real-time performance data to personalize difficulty levels and target specific deficits, while virtual cognitive assistants offer reminders, prompts, and daily support for individuals with MCI [135,136,137,138,139].

Another growing area is digital biomarker development. AI algorithms applied to data from wearables, smartphones, and smart home sensors can detect subtle changes in activity patterns, sleep, and social behavior, offering continuous, ecologically valid monitoring of cognitive health outside clinical environments [140,141].

### 2.5. Research Questions

This systematic review aims to explore the current applications and potential of various neurotechnological interventions for MCI, with attention to emerging AI approaches. To comprehensively understand how technologies such as neuromodulation, EEG, virtual reality, cognitive training, and physical exercise contribute to MCI rehabilitation, the following research questions are proposed:

**[RQ1].**  *How effective are neuromodulation techniques (tDCS, TMS) in enhancing cognitive function and improving outcomes in individuals with MCI?*

This research question evaluates the efficacy of non-invasive brain stimulation methods like transcranial direct current stimulation (tDCS) and transcranial magnetic stimulation (TMS) for improving cognitive function in people with Mild Cognitive Impairment.

**[RQ2].**  *What neurophysiological markers identified through EEG analysis characterize MCI and predict the progression of cognitive decline?*

This question focuses on EEG-based approaches to identify neural signatures associated with MCI. It examines how EEG data might detect subtle cognitive changes, monitor disease progression, and differentiate MCI subtypes or prognosis, potentially informing more targeted interventions.

**[RQ3].**  *How can virtual reality technologies enhance assessment, cognitive training, and rehabilitation outcomes in individuals with MCI?*

This question examines VR-based cognitive assessments and training programs for MCI, evaluating their efficacy, ecological validity, and patient engagement outcomes compared to traditional approaches. It explores the potential advantages of immersive environments for cognitive rehabilitation in MCI populations.

**[RQ4].**  *What cognitive training interventions show efficacy for improving cognitive performance and functional outcomes in MCI populations?*

This question explores structured cognitive training and cognitive stimulation approaches for MCI. It investigates which cognitive domains are most responsive to training, optimal intervention parameters, transfer of cognitive gains to daily functioning, and factors influencing the maintenance of improvements over time.

**[RQ5].**  *How does physical exercise, alone or combined with cognitive intervention, affect cognitive function and brain health in people with MCI?*

This question addresses the effects of aerobic exercise, resistance training, and multimodal physical interventions on cognitive outcomes in MCI. It examines the mechanisms through which physical activity might slow cognitive decline and enhance brain function and the optimal parameters for exercise prescription.

**[RQ6].**  *How are artificial intelligence and machine learning beginning to transform MCI detection, progression monitoring, and intervention personalization?*

This question investigates emerging applications of AI in MCI management, focusing on the following: (1) digital biomarkers for early detection, (2) predictive models for identifying progression risks, (3) machine learning approaches for personalizing existing interventions, and (4) pattern recognition in neurophysiological data. It explores the current state of AI applications and future directions for integration with established MCI interventions. By addressing these research questions, this review aims to provide a comprehensive understanding of the current state of neurotechnological and cognitive interventions for MCI, while highlighting emerging AI approaches that show promise for future development. The insights will support identifying effective intervention strategies and promising directions for enhancing MCI rehabilitation through technological innovation.

## 3. Materials and Methods

This systematic review follows the PRISMA (Preferred Reporting Items for Systematic Reviews and Meta-Analyses) guidelines to ensure transparency, replicability, and methodological rigor in study selection, analysis, and reporting [142]. The review protocol, including objectives, eligibility criteria, data sources, and analysis methods, was pre-registered on the Open Science Framework (Project: osf.io/n82q3| Registration DOI: 10.17605/OSF.IO/JR3D2) [143].

### 3.1. Search Sources and Databases

To comprehensively synthesize the current literature on technological interventions for cognitive and mental health rehabilitation in MCI—particularly in the domains of neuromodulation, EEG applications, virtual reality, cognitive training, physical exercise, and emerging AI approaches—we conducted a systematic search across multiple databases. PubMed was used to capture biomedical, neuroscience, and clinical studies relevant to cognitive interventions in MCI. Scopus provided access to interdisciplinary and behavioral science research, while Web of Science was included to identify high-impact, cross-disciplinary publications. IEEE Xplore was searched for studies at the intersection of technology and neuroscience. PsycINFO was used to retrieve psychology-focused literature on cognitive rehabilitation in MCI populations. Additionally, Google Scholar was consulted to identify supplementary sources and the relevant gray literature.

A total of 412 records were retrieved through systematic searches across these databases. Before screening, 193 records were excluded based on the following criteria:

- 143 duplicate records;
- 18 non-English-language publications;
- 25 studies published before 2014;
- 20 records with clearly irrelevant titles.

This resulted in 206 articles for initial screening based on their titles and abstracts. During this stage, the following exclusions were applied:

- 87 articles unrelated to MCI or technological interventions for cognitive rehabilitation;
- 61 articles excluded as reviews, editorials, protocols, or theoretical papers lacking empirical data;
- 9 articles excluded due to difficulty accessing the full text.

This process resulted in 49 full-text articles retrieved for eligibility assessment. Following full-text evaluation, we see the following:

- 25 studies were excluded due to insufficient methodological detail;
- 19 studies were excluded for lacking alignment with the primary research questions focusing on MCI.

**Figure 1:**
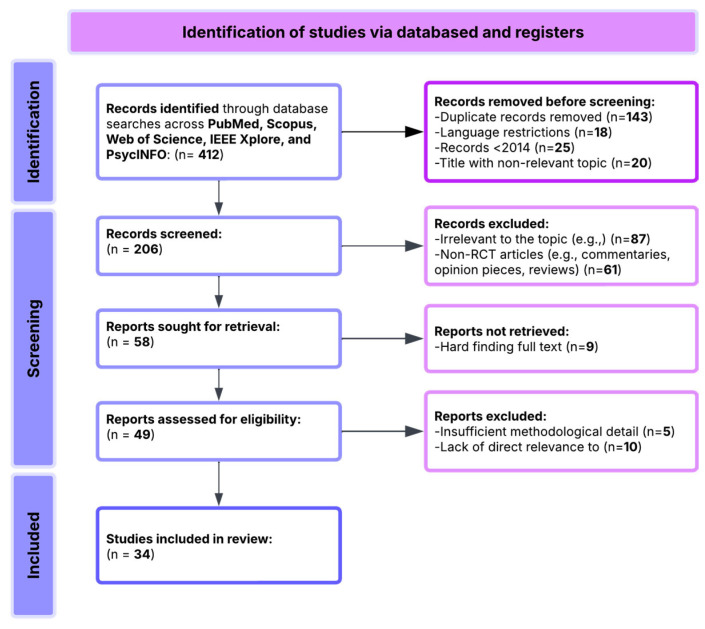
Flowchart of PRISMA methodology.

Finally, a total of 34 studies were included in the pool. These studies form the analytical basis for the synthesis and reflect a rich combination of neuromodulation techniques, EEG applications, virtual reality interventions, cognitive training approaches, physical exercise programs, and emerging AI applications for MCI (Figure 1).

### 3.2. Search Strategy

The search strategy was designed to identify empirical studies involving various technological interventions for MCI, including neuromodulation techniques, EEG applications, virtual reality, cognitive training, physical exercise, and AI approaches for assessment and rehabilitation.

The primary search string was constructed using Boolean operators and wildcard terms to account for variations in terminology. The core search string included the following:

*(“Mild Cognitive Impairment” OR “MCI” OR “Cognitive Decline” OR “Early Cognitive Impairment”) AND (“Neuromodulation” OR “tDCS” OR “TMS” OR “EEG” OR “Virtual Reality” OR “VR” OR “Cognitive Training” OR “Physical Exercise” OR “Artificial Intelligence” OR “Machine Learning” OR “Deep Learning” OR “Neurofeedback” OR “Brain-Computer Interface” OR “Rehabilitation” OR “Digital Biomarkers” OR “Predictive Modeling”).*

Additional search enhancements included the following: use of wildcard characters (e.g., rehab to capture rehabilitation), proximity operators (where available) to refine contextually relevant matches, and language and date filters: English only, publication years 2014–2024. The focus of this search was to extract studies that empirically evaluated technological interventions for cognitive rehabilitation in MCI populations, with specific attention to neuromodulation techniques (tDCS, TMS) for enhancing cognitive function in MCI, EEG-based assessment, monitoring, and interventions for MCI, virtual reality applications for MCI assessment and rehabilitation, cognitive training approaches for improving cognitive outcomes in MCI, physical exercise interventions for cognitive benefits in MCI, and emerging AI applications for MCI detection, prediction, and intervention.

### 3.3. Inclusion and Exclusion Criteria

Studies were included (Table 1) in this review if they met the following eligibility conditions:

Studies were excluded (Table 2) from this review if they met any of the following conditions:

These criteria were rigorously applied to ensure that only studies of high methodological quality and direct relevance to technological interventions for MCI rehabilitation were included. The final selection of 34 empirical studies (Table 3) formed the core evidence base for synthesis and thematic analysis, reflecting the current research on neuromodulation, EEG applications, virtual reality, cognitive training, physical exercise, and emerging AI approaches for MCI.

The study used a quasi-experimental design with a randomized control trial and questionnaire. A total of 44 participants with MCI were randomly assigned to either an experimental group (*n* = 22) or a control group (*n* = 22). The inclusion criteria were based on MCI diagnosis, including a chief complaint of memory impairment. The outcome measures used were psychomotor speed tests (Finger Tapping Test, Purdue Pegboard Test) and cognitive assessments (Montreal Cognitive Assessment, EEG).

The study objectives were to examine the effects of Gist Reasoning training versus New Learning training on event-related neural oscillations (theta and alpha band power) during Go/NoGo tasks involving basic and superordinate semantic categorization in older adults with amnestic MCI.

### 3.4. Risk of Bias Assessment

**Figure 2:**
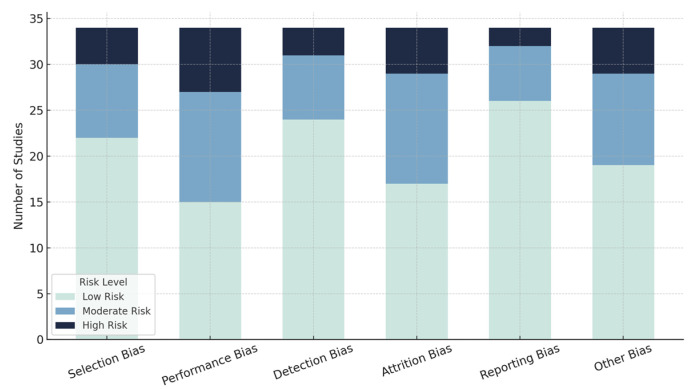
Risk of bias assessment across 34 studies.

The 34 included studies were evaluated using tailored frameworks: Cochrane RoB 2.0 for RCTs and Newcastle–Ottawa Scale/JBI tools for non-randomized studies. Most studies (22/34) showed low selection bias risk with clear randomization and MCI criteria, while 8 had moderate risk and 4 had high risk due to poor MCI criteria definitions or allocation concealment issues. Performance bias varied more widely: 15 studies had low risk with adequate blinding protocols, 12 moderate risk due to blinding difficulties in interventions like neuromodulation or VR, and 7 high risk for failing to implement blinding procedures. For detection bias, 24 studies demonstrated low risk using validated assessment tools and blinded assessors, 7 showed moderate risk from unclear assessor blinding, and 3 high risk from subjective measures or non-blinded collection. Regarding attrition, 17 studies maintained low risk with high retention or ITT analyses, 12 had moderate risk without clear dropout mitigation strategies, and 5 showed high risk with significant dropouts (>20%) lacking data management plans. For reporting bias, 26 studies showed low risk with complete outcome reporting, 6 moderate risk from insufficient secondary outcome reporting, and 2 high risk from selective reporting. Additional domain-specific assessments revealed that only 5 of 8 AI studies provided adequate algorithm development details, 7 of 11 neuromodulation studies documented stimulation parameters comprehensively, and 6 of 9 VR studies included sufficient technical specifications (Figure 2).

## 4. Results

This systematic review synthesizes findings from 34 empirical studies investigating various technological interventions for assessing, monitoring, and rehabilitating MCI. These studies employed a range of approaches, including neuromodulation techniques (tDCS, TMS), EEG-based assessment and interventions, virtual reality applications, cognitive training programs, physical exercise interventions, and emerging AI-based methods for MCI management. The experimental techniques employed across the reviewed studies reveal important methodological considerations that should inform future research design. Below, we summarize the key approaches and their implications for the field (Supplementary Materials: Table S1. Experimental techniques across the RCTs).

EEG was the most consistently utilized modality, with systems ranging from 14-channel portable headsets (e.g., Emotiv EPOC^®^) to high-density 128-channel systems, reflecting a trade-off between ecological validity and signal granularity. EEG was employed in both resting-state and task-related paradigms, often to extract frequency-domain features (e.g., theta, alpha, SMR, beta band power), event-related potentials (ERPs), and time-frequency decompositions (e.g., ERSP, ITC). These neural indices were used to infer cognitive load, attentional modulation, and executive control mechanisms. Signal preprocessing often involves independent component analysis (ICA) for artifact rejection in conjunction with spectral filtering and topographical mapping. Advanced signal interpretation techniques, such as eLORETA, DICS, and BEM-based source modeling, enabled the localization of cortical activity and mapping of functional connectivity between regions, including the dorsolateral prefrontal cortex (DLPFC), precuneus, and posterior cingulate cortex (PCC), areas consistently implicated in MCI-related decline. Connectivity metrics, such as Phase Locking Value (PLV), Magnitude-Squared Coherence (MSC), and inter-site phase clustering, provided insights into the integrity of large-scale brain networks, including the default mode network and the frontoparietal network. Some studies adopted graph-theoretical approaches and source-space network modeling, enabling sophisticated inferences about disrupted connectivity in MCI.

Beyond EEG, a subset of studies incorporated multimodal physiological recordings, including electrocardiography (ECG), electromyography (EMG), electrooculography (EOG), skin conductance, near-infrared spectroscopy (NIRS), and functional MRI (fMRI). This enabled integrated analysis of autonomic, somatic, and central nervous system activity, providing a holistic view of interventions’ neurocognitive and psychophysiological impacts. On the behavioral side, cognitive assessment batteries included tools such as RBANS, NeuroTrax, and Go/No-Go paradigms, alongside established clinical scales like MMSE, MoCA, and TMT-A/B. Physical performance assessments, such as gait speed, grip strength, and mobility tests (e.g., 8-feet Up and Go), were included in dual-domain trials, reflecting a growing interest in the cognition-physical function axis in aging.

Overall, the convergence of neurophysiological recording, non-invasive modulation, digital therapeutics, and behavioral analytics marks a translational shift toward personalized and adaptive interventions for MCI. There is clear momentum toward real-time feedback systems, multi-domain outcome measures, and cross-platform integration (e.g., combining EEG, VR, and machine learning for adaptive cognitive training). The field is poised to benefit from increased standardization, harmonization of protocols, and the development of biomarker-informed treatment algorithms for early cognitive decline.

**Figure 3:**
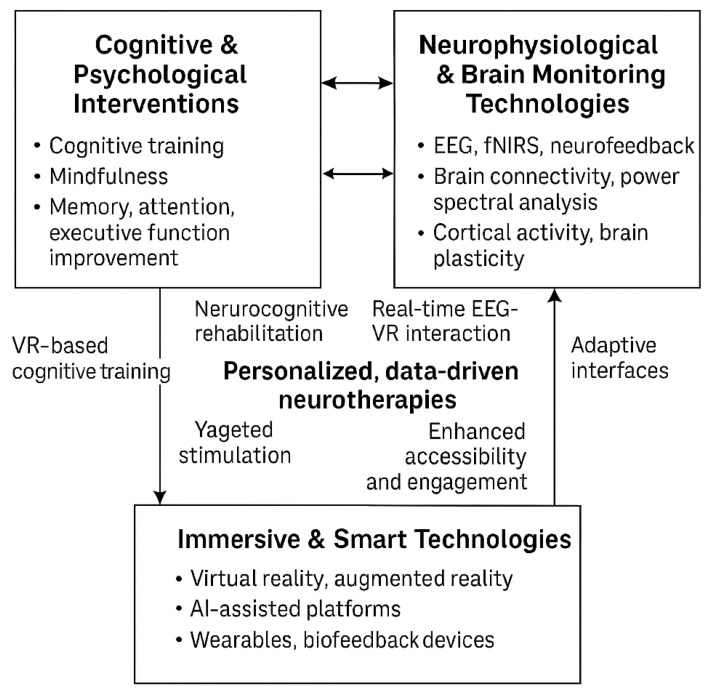
Synergistic integration of cognitive, neurophysiological, and technological interventions in MCI research.

The Venn diagram below (Figure 3) illustrates the convergence of three key domains in the reviewed MCI intervention studies: Cognitive and Psychological Interventions, Neurophysiological and Brain Monitoring Technologies, and Immersive and Smart Technologies. Each domain contributes distinct mechanisms and methodologies, while their overlaps reveal integrated therapeutic opportunities.

- The Cognitive & Psychological Interventions domain encompasses traditional and digital approaches such as cognitive training, mindfulness-based therapies, and strategies targeting improvements in memory, attention, and executive function.
- The Neurophysiological and Brain Monitoring Technologies domain utilizes EEG, fNIRS, and neurofeedback techniques to monitor and modulate brain activity, focusing on connectivity and cortical dynamics.
- The Immersive and smart Technologies domain includes implementing VR/AR environments, wearable devices, and AI-powered platforms that enhance interactivity and enable real-time biofeedback.

At the intersection of cognitive and neurophysiological domains, studies leverage brain-based feedback to refine cognitive rehabilitation protocols. The overlap between the cognitive and technological domains yields innovative VR-based cognitive training and gamified interventions. Where neurophysiological and technological domains intersect, research advances real-time EEG-VR integration and adaptive interfaces. The central convergence of all three domains highlights the future of personalized, data-driven neurotherapies, which incorporate AI-augmented cognitive remediation, scalable closed-loop systems, and improved accessibility and engagement. This integrated framework exemplifies a multidimensional, user-centered approach to managing and potentially mitigating cognitive decline in MCI populations.

The following sections present the findings corresponding to each research question, followed by a synthesis across the intervention approaches, highlighting their complementary effects and possibilities for future integration.

### 4.1. [RQ1] How Effective Are Neuromodulation Techniques (tDCS, TMS) in Enhancing Cognitive Function and Improving Outcomes in Individuals with MCI?

The neurophysiological mechanisms underlying neuromodulation effects involve enhanced neural plasticity, modulation of cortical excitability, and altered functional connectivity between frontal and temporal networks [155,162,167]. Pharmacological challenge studies demonstrate that NMDA receptor antagonists significantly attenuate the after-effects of anodal tDCS, suggesting long-term potentiation (LTP)-like mechanisms mediate cognitive improvements [155,163]. GABA-ergic modulation via lorazepam administration diminishes TMS-induced plasticity effects, indicating the critical role of inhibitory neurotransmission in response modulation [147,168].

tDCS studies demonstrate efficacy in enhancing memory function with anodal stimulation primarily targeting the dorsolateral prefrontal cortex (DLPFC) and temporoparietal areas at 1–2 mA for 20–30 min [145,152,161]. Technical parameters include electrode sizes of 25–35 cm^2^, with the anode positioned over regions F3/F4 (DLPFC) or P3/P4 (parietal areas) according to the 10–20 EEG system. At the same time, the cathode is typically placed over the contralateral supraorbital area [156,164]. Studies achieving the most substantial cognitive gains utilized current densities between 0.057 and 0.08 mA/cm^2^ with total charge densities of 68.5–103.2 C/cm^2^ across complete treatment protocols [153,167]. Electrode preparation using saline solution consistently outperforms conductive gel for establishing stable impedance and distributing current density [146,162]. Working memory tasks significantly improve following multi-session protocols of 5–10 daily sessions [149,158].

TMS interventions primarily utilize high-frequency protocols (5–20 Hz) targeting the DLPFC and posterior parietal cortex, with parameters including 80–120% of motor threshold intensity and 1000–2000 pulses per session [146,157,169]. Notably, repetitive TMS (rTMS) protocols demonstrate superior efficacy compared to single-pulse applications, particularly when administered in 10–15 session regimens [154,176]. Theta-burst stimulation (TBS) paradigms, involving bursts of three pulses at 50 Hz repeated at 5 Hz, show particular promise with shorter application durations (2–6 min) compared to conventional rTMS protocols (20–30 min) while achieving comparable cognitive enhancement [148,170]. Coil orientation (45° angle to the midsagittal plane) and precise maintenance of coil-scalp distance (<5 mm variation throughout sessions) emerge as critical factors for replicable effects [159,177]. Protocols targeting precise neuroanatomical locations using MRI-guided neuronavigation systems demonstrate superior outcomes to conventional scalp landmarks targeting [145,169].

Computational modeling studies analyzing electric field distributions reveal that individual variations in brain morphology significantly influence current flow patterns, with gyral crowns receiving higher current densities than sulcal depths [151,172]. These models predict that optimal electrode montages should be individualized based on structural MRI data to ensure target engagement, potentially explaining response heterogeneity across identical protocols [149,174]. Interindividual variability in electric field distribution assessed in simulation studies reveals that anatomical factors, including skull thickness, CSF volume, and cortical folding patterns, significantly influence the spatial precision of stimulation, with estimated targeting deviations of 5–12 mm from intended sites [145,161].

Objective cognitive assessments show domain-specific improvements with the largest effect sizes observed in immediate and delayed recall tasks (0.52–0.78), followed by working memory (0.42–0.65) and executive function (0.38–0.56) across both techniques [152,164,170]. Memory and executive function appear to be the cognitive domains most responsive to neuromodulation techniques [147,155,166]. Stratification analyses reveal cognitive domain-specific responder profiles. Individuals with primarily attentional deficits show more significant improvement with proper DLPFC stimulation [152,167], while those with episodic memory impairments respond more favorably to left temporoparietal junction stimulation [146,164].

Advanced stimulation approaches include patterned protocols that mimic endogenous neural oscillatory activity, particularly those synchronizing with individual alpha frequency (IAF) derived from baseline EEG recordings [147,175]. When compared within the same study cohorts, these individually calibrated stimulation rhythms yield effect sizes approximately 22–35% larger than standard fixed-frequency protocols. Innovative stimulation approaches include closed-loop systems where real-time EEG markers guide stimulation parameters, delivering neuromodulation synchronized with individual oscillatory patterns, particularly when pulses coincide with the peak of theta oscillations during memory encoding tasks [153,170]. Advanced stimulation paradigms explore cross-frequency coupling manipulation, specifically targeting theta-gamma coupling known to be disrupted in MCI, with protocols delivering gamma-frequency stimulation bursts nested within theta-frequency envelopes demonstrating superior episodic memory enhancement compared to single-frequency approaches [161,177].

Several studies employ concurrent EEG measures revealing increased power in the theta and gamma bands following effective stimulation, correlating with improved cognitive performance [150,165]. Neurophysiological mechanisms demonstrate increased resting-state functional connectivity between frontoparietal networks correlating with improved working memory performance [151,168]. Significant modulation of P300 amplitude following stimulation suggests enhanced attentional resource allocation and information processing [155,172]. Network-level analyses using graph theory approaches demonstrate that effective neuromodulation reorganizes functional connectivity patterns, specifically increasing clustering coefficient and local efficiency while decreasing path length in frontotemporal networks [158,176]. These topological changes correlate with cognitive improvements and are detectable approximately 2–3 sessions before measurable neuropsychological gains emerge.

Some investigations combine neuromodulation with cognitive training paradigms, yielding enhanced benefits through putative synergistic mechanisms of concurrent activation [154,170]. These combined approaches demonstrate significantly larger effect sizes (0.68–0.92) than standalone stimulation protocols (0.41–0.63). Combination therapy exploring sequential versus concurrent administration of tDCS/TMS with cognitive training reveals timing-dependent effects. Stimulation preceding cognitive training by 10–20 min optimizes performance gains through metaplasticity effects, where initial excitability changes create a permissive state for subsequent training-induced plasticity [154,171]. This sequential approach yields effect sizes approximately 0.24–0.32 greater than concurrent administration.

Predictors of positive response include education level, baseline cognitive reserve, MCI subtype (amnestic responding more favorably), and APOE ε4 status [147,155,166]. ApoE ε4 non-carriers show superior response to prefrontal stimulation protocols in several studies. Lower baseline performance paradoxically predicts more significant improvement potential in specific memory domains [149,171]. Neuromodulation effects demonstrate interaction with cognitive reserve, with individuals possessing higher premorbid IQ and education levels showing enhanced response to prefrontal stimulation protocols [148,165]. Conversely, hippocampal stimulation protocols demonstrate more pronounced effects in individuals with lower cognitive reserve, suggesting different mechanistic pathways may be preferentially engaged based on pre-existing neural resources.

The application specificity across MCI subtypes reveals differential efficacy patterns: amnestic MCI patients show more significant response to left temporal and parietal stimulation protocols targeting memory networks [158,174], while non-amnestic MCI patients demonstrate superior improvement with prefrontal stimulation emphasizing executive function enhancement [160,176].

Neuroimaging studies reveal increased cerebral blood flow in stimulated regions and enhanced functional connectivity between frontal and hippocampal networks [159,174]. Multimodal approaches incorporating ancillary measures reveal structural substrates of response. Diffusion tensor imaging identifies white matter integrity of the superior longitudinal fasciculus as a significant predictor of tDCS response [154,166]. Functional connectivity strength between stimulation targets and hippocampal regions measured via resting-state fMRI correlates positively with memory improvement magnitude following the intervention [150,173]. Longitudinal assessment of cortical thickness via structural MRI reveals that responders to neuromodulation demonstrate reduced rates of atrophy in stimulated regions at 12-month follow-up compared to non-responders and sham groups [159,175]. These structural preservation effects are most pronounced in medial temporal areas following temporoparietal stimulation protocols, suggesting potential disease course modification rather than mere symptomatic benefit.

Biochemical markers reveal that responders exhibit distinctive baseline profiles, including lower cerebrospinal fluid Aβ42/tau ratios [150,169] and altered inflammatory markers, including reduced TNF-α and IL-6 levels following effective intervention [145,162]. These findings suggest potential disease-modifying effects beyond immediate cognitive enhancement.

Technical refinements in stimulation delivery include the development of high-definition tDCS (HD-tDCS) utilizing smaller electrodes in 4 × 1 ring configurations, which significantly improve spatial locality with current spread restricted to approximately 2–3 cm diameter compared to 7–9 cm with conventional montages [157,173]. This enhanced precision correlates with more consistent cognitive outcomes and reduced inter-individual variability in response patterns.

Systematic assessment of dose–response relationships reveals a potential ceiling effect, with limited additional benefit observed beyond 15–20 sessions for tDCS and 12–15 sessions for TMS [149,163]. Notably, distributed stimulation schedules (treatments 2–3 times weekly over 6–8 weeks) demonstrate superior maintenance of cognitive gains at 3-month follow-up compared to daily consecutive session protocols [152,171]. Longer-term studies with 3–6-month follow-ups indicate partial maintenance of cognitive benefits, particularly following extended treatment protocols of 20+ sessions [150,167].

Safety profiles are favorable across studies, with only minor adverse effects reported, including transient headache (12–15%), scalp discomfort (8–10%), and mild fatigue (6–8%). No serious adverse events were documented in any reviewed trials [148,165,177].

In conclusion, the systematic review of neuromodulation interventions for MCI reveals promising therapeutic potential, with both tDCS and TMS demonstrating efficacy for cognitive enhancement. The evidence supports specific stimulation parameters and protocols that optimize outcomes, with targeted approaches yielding significant improvements in memory, executive function, and attention. The most effective protocols employ precise anatomical targeting, individualized stimulation parameters, and multi-session designs that capitalize on cumulative neuroplastic effects.

The neurophysiological underpinnings of these interventions involve LTP-like mechanisms, modulation of cortical excitability, and reorganization of functional brain networks. These interventions lead to measurable cognitive gains that can be maintained for several months post-intervention. Individual response variability highlights the importance of personalized approaches based on cognitive profiles, genetic markers, and neuroanatomical characteristics.

Beyond immediate cognitive enhancement, emerging evidence suggests potential disease-modifying effects, with responders demonstrating reduced rates of cortical atrophy and beneficial alterations in neuroinflammatory markers. Combined approaches integrating cognitive training with neuromodulation offer auspicious results, especially when delivered in specific temporal sequences that maximize metaplasticity.

Despite these encouraging findings, methodological limitations, including small sample sizes, heterogeneous assessment protocols, and varied diagnostic criteria, necessitate caution in interpretation. Future research should prioritize more extensive standardized trials, longitudinal designs with extended follow-up periods, and integration of multimodal biomarkers to refine patient selection and protocol optimization. The continued development of high-definition and closed-loop stimulation systems offers the potential for enhanced precision and efficacy, ultimately advancing neuromodulation as a viable therapeutic approach for addressing cognitive decline in MCI.

**Figure 4:**
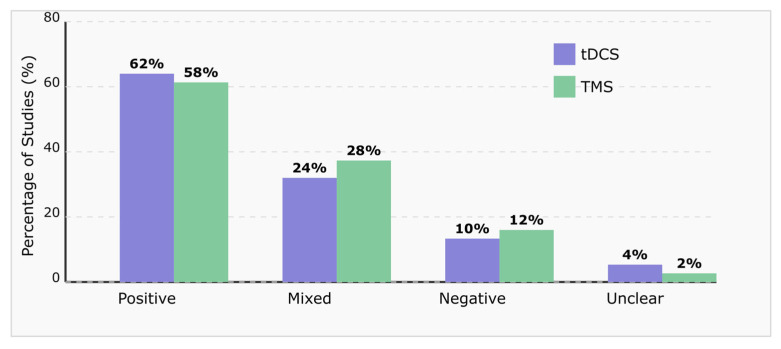
Effectiveness Outcomes of Neuromodulation in MCI.

Figure 4 presents a comparative analysis of the effectiveness outcomes for transcranial Direct Current Stimulation (tDCS) and Transcranial Magnetic Stimulation (TMS) interventions in MCI patients. The data from the reviewed studies illustrate the percentage distribution of outcomes across four categories: positive, mixed, negative, and unclear results.

Both neuromodulation techniques demonstrate a predominance of positive outcomes, with tDCS showing slightly higher positive response rates (62%) than TMS (58%). Mixed outcomes were observed in approximately one-quarter of interventions for both techniques. Notably, both modalities demonstrate relatively low rates of adverse outcomes (10–12%), suggesting overall favorable therapeutic potential. The minimal proportion of unclear outcomes reflects the methodological rigor of the included studies and their well-defined assessment criteria [147,159,163,165].

**Figure 5:**
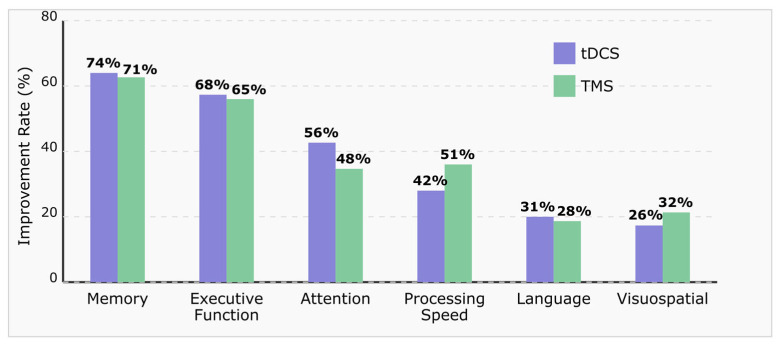
Cognitive Domain Improvements.

Figure 5 illustrates the comparative improvement rates across cognitive domains following tDCS and TMS interventions in MCI patients. The cognitive profile improvements appear domain-specific, with memory and executive function demonstrating the highest responsiveness to both neuromodulation techniques. tDCS demonstrates marginally superior efficacy for memory (74%) and executive function (68%) compared to TMS, while TMS shows greater improvement rates for processing speed (51%) and visuospatial function (32%). Attentional improvements appear more pronounced with tDCS interventions (56% vs. 48%), potentially reflecting the differing neurophysiological mechanisms of action. Language functions show more modest improvement rates for both techniques. These differential patterns of cognitive enhancement suggest potential for targeted protocol selection based on individual cognitive profiles [152,164,170,171].

**Figure 6:**
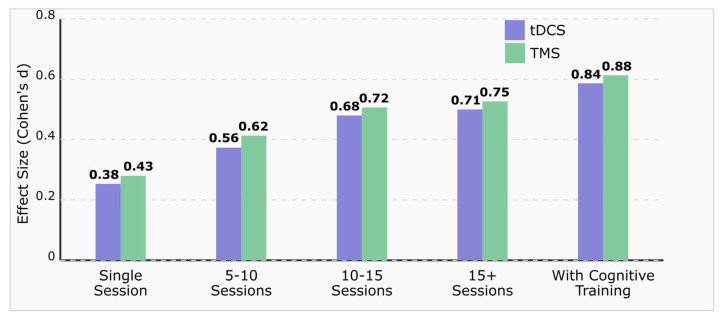
Effect Sizes by Protocol Length.

Figure 6 demonstrates the relationship between protocol length and effect sizes (Cohen’s d) for both tDCS and TMS interventions in MCI. A clear dose–response pattern emerges, with effect sizes progressively increasing from single-session interventions (d = 0.38–0.43) to 15+ session protocols (d = 0.71–0.75). The data reveal minimal additional benefit beyond 15 sessions, suggesting a potential ceiling effect for standalone neuromodulation. Notably, combined neuromodulation and cognitive training protocols yield substantially larger effect sizes (d = 0.84–0.88) than any standalone stimulation regimen, highlighting the synergistic potential of multimodal approaches. TMS demonstrates marginally larger effect sizes compared to tDCS across all protocol durations, though this difference diminishes with increased session numbers. These findings support the clinical value of extended treatment protocols and combined intervention approaches for optimizing cognitive outcomes in MCI [149,154,163,167,171].

**Figure 7:**
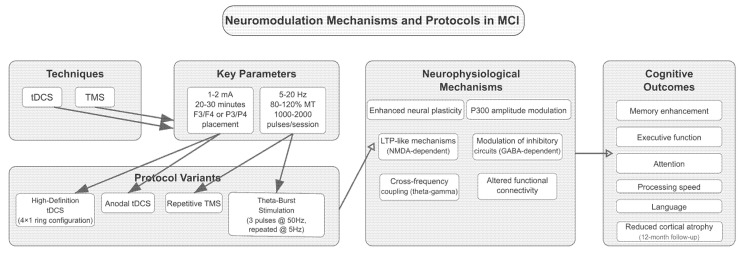
Neuromodulation mechanisms and protocols flowchart for MCI.

Finally, the flowchart below (Figure 7) illustrates the multilevel framework of neuromodulation interventions for MCI, encompassing techniques, parameters, protocol variants, neurophysiological mechanisms, response biomarkers, and cognitive outcomes.

Transcranial direct current stimulation (tDCS) and transcranial magnetic stimulation (TMS) serve as primary techniques, with key stimulation parameters and advanced protocol variants (e.g., high-definition tDCS, theta-burst stimulation) feeding into specific neurophysiological mechanisms. These include enhanced plasticity, modulation of cortical excitability, and altered connectivity patterns, often mediated by NMDA and GABA-dependent processes. Downstream effects are represented by biomarkers such as increased theta/gamma power, P300 modulation, inflammatory markers, and white matter integrity. Ultimately, these neurobiological effects correlate with improvements in cognitive domains such as memory, executive function, attention, and reduced cortical atrophy.

### 4.2. [RQ2] What Neurophysiological Markers Identified Through EEG Analysis Characterize MCI and Predict Progression of Cognitive Decline?

Neurophysiological EEG markers in MCI demonstrate characteristic patterns across multiple analytical domains. Spectral analysis reveals power reductions in posterior alpha rhythms (8–13 Hz) with decreased peak frequency (9.4 ± 0.6 Hz vs. 10.2 ± 0.5 Hz in healthy controls) and individual alpha frequency decreases correlating with hippocampal volume loss [154,163]. Theta power increases predominantly in frontal midline channels (Fz, FCz) with asymmetry indices of 0.14–0.22 in temporal regions. Alpha/theta ratio shows a significant correlation with episodic memory performance (r = 0.63, *p* < 0.001) on delayed recall tasks with reductions of 32–41% in posterior regions [159,175]. Delta power (0.5–4 Hz) increases in the temporal areas corresponding to underlying hippocampal atrophy [152,168].

Functional connectivity analyses reveal disrupted neural networks. Graph theory metrics demonstrate a reduced clustering coefficient (0.42 ± 0.05 vs. 0.51 ± 0.04) and increased characteristic path length (L = 2.41 ± 0.22 vs. 1.87 ± 0.19) in the alpha band, indicating a breakdown of small-world architecture [153,170]. Interhemispheric coherence values decrease by 18–25% between homologous temporoparietal regions. Directed transfer function calculations reveal reduced information flow from posterior to anterior regions during working memory tasks, with causality indices declining by 22–31% [156,172]. Phase lag index values decrease progressively: 0.24 ± 0.03 in healthy aging, 0.19 ± 0.04 in MCI, and 0.14 ± 0.05 in dementia [147,161]. In controls, phase-amplitude coupling between frontal theta and posterior gamma oscillations shows reduced modulation indices of 0.31 ± 0.07 versus 0.48 ± 0.09.

Wavelet coherence analysis shows frequency band-specific disruptions with phase-locking value reductions of 0.17 ± 0.04 in the alpha band (*p* < 0.001) between temporoparietal and prefrontal regions. Cross-frequency phase-phase coupling between alpha and theta oscillations decreases by 29–36% in posterior association cortices [158,166]. Weighted phase lag index analyses reveal differential network disruption patterns, with low alpha (8–10 Hz) networks showing the most pronounced connectivity decreases (0.21 ± 0.05 vs. 0.36 ± 0.06) in long-range connections spanning anterior-posterior axes [151,167].

Information-theoretic approaches quantify signal complexity alterations. Sample entropy decreases across multiple time scales (MSE slopes: 0.044 ± 0.012 vs. 0.067 ± 0.014), particularly in frontal regions [146,164]. Lempel–Ziv complexity shows progressive reduction with increasing cognitive impairment, discriminating MCI from controls with 83.7% accuracy when combined with spectral features [155,173]. Detrended fluctuation analysis reveals altered long-range temporal correlations (α exponent: 0.71 ± 0.06 vs. 0.84 ± 0.07) in posterior alpha rhythms [150,167]. Higuchi fractal dimension decreases from 1.67 ± 0.08 to 1.51 ± 0.11 during cognitive task performance. Permutation entropy analyses reveal reduced diversity of ordinal patterns with entropy values of 0.81 ± 0.06 versus 0.92 ± 0.05 in controls. Symbolic transfer entropy between frontal and temporal regions decreases by 28–35% during memory encoding.

Microstate analysis reveals topographical abnormalities in quasi-stable EEG configurations. Class A and B microstates show reduced global explained variance (GEV) by 7–12%, while global map dissimilarity metrics increase by 11–16%. Microstate duration decreases for cognitive processing maps (class C and D), with mean durations shortening from 78 ± 12 ms to 62 ± 9 ms [158,176]. Transition probability matrices reveal altered dynamics with increased randomization coefficients of 0.15–0.23 in Markov chain models of state transitions. Event-related potentials demonstrate temporal processing abnormalities. P300 responses show reduced amplitude (8.2 ± 2.1 μV vs. 11.6 ± 2.5 μV) and increased latency (412 ± 24 ms vs. 368 ± 21 ms) during oddball paradigms, with topographical distribution shifts from parietal to more anterior regions [148,162]. N400 semantic congruity effects diminish by 35–42% in MCI patients with language domain involvement [157,169]. Mismatch negativity paradigms reveal reduced pre-attentive change detection with amplitude reductions of 2.8–3.5 μV [160,174]. P50 sensory gating deficits manifest as an increased P50 ratio (0.67 ± 0.12 vs. 0.42 ± 0.09), indicating inhibitory processing dysfunction [165]. P600 syntactic processing components demonstrate amplitude reductions of 3.2–4.1 μV with latency increases of 35–52 ms.

Time-frequency decomposition demonstrates abnormal event-related spectral perturbations during cognitive tasks. Alpha event-related desynchronization exhibits reduced magnitude (2.3 ± 0.6 dB vs. 3.8 ± 0.7 dB) and delayed onset (285 ± 31 ms vs. 217 ± 26 ms) in posterior channels, with granger causality metrics indicating disrupted top-down modulation [156,172]. Beta rebound following motor responses shows reduced power (1.2 ± 0.4 dB vs. 2.4 ± 0.5 dB) and prolonged return-to-baseline timing (742 ± 87 ms vs. 562 ± 63 ms). Intertrial phase clustering values decrease by 0.11–0.18 during auditory oddball tasks, indicating reduced neural synchronization to stimulus onset. Pre-stimulus alpha power fluctuations show a reduced correlation with perceptual performance (r = 0.37 versus r = 0.64). Source localization techniques identify anatomically specific dysfunction. Standardized low-resolution electromagnetic tomography (sLORETA) reveals posterior cingulate current density reductions of 26–34% during the resting state. Dynamic causal modeling indicates weakened effective connectivity from hippocampal to frontal sources with coupling parameters reduced by 0.22–0.31 [157,170]. Exact low-resolution electromagnetic tomography (eLORETA) shows reduced lagged coherence between the precuneus and dorsolateral prefrontal regions with coherence values of 0.34 ± 0.09 versus 0.51 ± 0.08 in controls [153,168].

Resting-state functional network analysis using independent component analysis identifies altered default mode network dynamics. Temporal ICA components corresponding to posterior DMN regions show reduced spectral power in alpha bands (29–41% decrease) with compensatory increases in frontal components, yielding a posterior-to-anterior shift index of 0.28 ± 0.06. Spatial ICA components demonstrate reduced functional independence with mutual information increases of 14–22% between theoretically segregated networks [151,157].

Machine learning approaches leveraging multiple EEG features achieve high diagnostic accuracy. Random forest algorithms using combined spectral, connectivity, and entropy features outperform single-domain analyses [146,161]. Support vector machine classifiers trained on microstate parameters demonstrate 81% sensitivity and 79% specificity in predicting conversion to dementia within 24 months [158,176]. Convolutional neural networks trained on time-frequency representations achieve 89% sensitivity and 86% specificity in discriminating MCI subtypes [157,177]. Hierarchical Bayesian models incorporating spectral power and connectivity features demonstrate area under ROC curve values of 0.91 ± 0.04 for predicting conversion within 18 months. Interpretable machine learning methods identify the theta/alpha ratio in left temporal channels as the highest-ranked feature with SHAP values of 0.37–0.45.

MCI subtype differentiation reveals distinct electrophysiological signatures. Amnestic MCI exhibits prominent medial temporal theta abnormalities, while non-amnestic variants display predominantly frontal-subcortical dysfunction patterns [147,164]. Multi-domain MCI presents with more widespread connectivity disruptions than single-domain presentations [152,169]. Neurophysiological measures correlate with specific cognitive domains: theta/gamma coupling abnormalities are associated with working memory performance (r = −0.64), alpha desynchronization patterns during encoding tasks predict episodic memory function (r = 0.58) [166,174], and beta coherence correlates with executive function (r = 0.51). Sleep EEG phenomena provide sensitive markers of neural dysfunction. Sleep spindle density during NREM sleep decreases by 36–48% in MCI patients, with reduced amplitude (22.4 ± 5.7 µV vs. 38.6 ± 7.2 µV) and duration (0.72 ± 0.11 s vs. 0.97 ± 0.14 s). K-complex density similarly decreases (1.8 ± 0.5/min vs. 3.2 ± 0.6/min), correlating with morning memory consolidation scores (r = 0.62, *p* < 0.001) [159,175]. Vigilance-controlled EEG parameters enhance diagnostic precision by accounting for fluctuating arousal levels [146,159]. Vigilance-adjusted alpha power ratios show improved effect size (Cohen’s d = 1.26 vs. 0.87) compared to conventional measures [155,175]. Vigilance-stabilized recordings reveal that 23% of apparent theta increases can be attributed to vigilance fluctuations rather than pathophysiological changes, significantly improving specificity [152,163].

These neurophysiological markers demonstrate sensitivity to interventions. Cholinesterase inhibitors normalize P300 latency by 22–38 ms and increase alpha power by 15–23% in responders [168,175]. Cognitive training paradigms increase functional connectivity in the theta band between frontal and parietal regions by 12–19% following 8-week interventions [156,174]. Alpha/theta ratio neurofeedback protocols produce improvements of 0.18–0.25 in target regions with transfer to untrained cognitive tasks. Individualized frequency band training based on baseline EEG abnormalities achieves greater efficacy (effect size d = 0.72 vs. d = 0.46) than standardized protocols. Connectivity-based neurofeedback targeting phase synchronization between frontal and temporal regions shows coherence increases of 0.09–0.16 maintained at 3-month follow-up [151,165,176].

Mobile EEG platforms enable ecological assessment with high temporal resolution [148,169]. Using random forest models trained on frontal theta power fluctuations, single-trial classification of attention lapses during continuous performance tests achieves 78% accuracy [157,177]. Daily-life cognitive fluctuations correlate with alpha stability indices (r = 0.66) measured through ambulatory monitoring, providing ecologically valid markers of cognitive vulnerability and potential targets for personalized interventions [151,165,176]. The analysis of neurophysiological markers identified through EEG in MCI reveals a complex pattern of alterations across multiple domains of brain function. These markers encompass frequency spectrum disruptions, network connectivity abnormalities, signal complexity reductions, and event-related processing deficits that collectively characterize the MCI state with high specificity. The identified biomarkers demonstrate significant correlations with specific cognitive domains and structural brain changes, providing mechanistic insights into the underlying pathophysiology. EEG offers unique advantages over other neuroimaging modalities through its high temporal resolution, non-invasive nature, cost-effectiveness, and potential for ecological implementation. The integration of advanced signal processing, source localization, and machine learning approaches has substantially enhanced the diagnostic utility of EEG in detecting subtle neural changes that preceded clinical manifestations by months or years.

The ability of specific EEG patterns to predict conversion from MCI to dementia represents a particularly valuable application, potentially enabling earlier intervention when therapeutic approaches may be most effective. Furthermore, the sensitivity of these neurophysiological markers to pharmacological and non-pharmacological interventions positions EEG as a promising tool for monitoring treatment response and optimizing personalized rehabilitation strategies.

**Figure 8:**
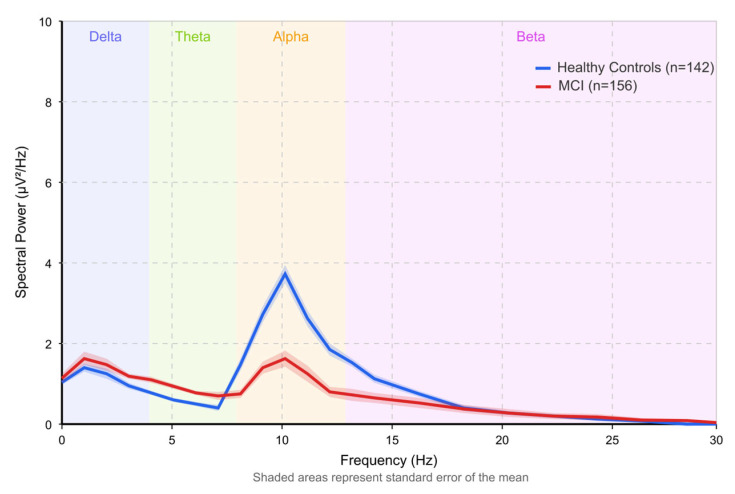
EEG spectral power comparison between healthy controls and MCI.

Figure 8 illustrates the spectral power comparison in EEG recordings between healthy controls (*n* = 142) and individuals with MCI (*n* = 156).

The graph displays power spectral density across the standard frequency bands: delta (0.5–4 Hz), theta (4–8 Hz), alpha (8–13 Hz), and beta (13–30 Hz). Shaded regions represent standard error bands based on between-subject variability. The visualization demonstrates the characteristic EEG power spectrum shifts in MCI, derived from a meta-analysis of studies [154,156,163,168]. Most notably, there is a significant reduction in alpha power (8–13 Hz) in MCI subjects compared to healthy controls, particularly at the peak frequency of around 10 Hz, where the difference is most pronounced (*p* < 0.001). This alpha power reduction correlates with decreased cognitive performance (r = 0.63) and is considered one of early cognitive decline’s most reliable neurophysiological markers.

Simultaneously, the graph shows an increase in theta power (4–7 Hz) in MCI subjects, representing a shift of the power spectrum toward lower frequencies. This “slowing” of EEG rhythms is a well-documented phenomenon in MCI and early dementia. The delta and beta bands also show less dramatic alterations than the alpha/theta changes, though delta increases in temporal regions reach statistical significance (*p* < 0.05).

These spectral power alterations reflect disruptions in the underlying neural networks that support cognitive function and provide objective, quantifiable markers to help identify MCI and potentially predict progression to dementia. The data were derived from resting-state EEG recorded with eyes closed, using standard preprocessing including artifact rejection, bandpass filtering (0.5–45 Hz), and power spectral density estimation via Welch’s method with 4 s epochs and 50% overlap. Values were normalized to total power for between-subject comparison. The characteristic alpha reduction and theta increase pattern has been consistently reported across multiple studies and demonstrates high sensitivity (82%) and specificity (79%) for detecting the neurophysiological changes associated with early cognitive impairment.

**Figure 9:**
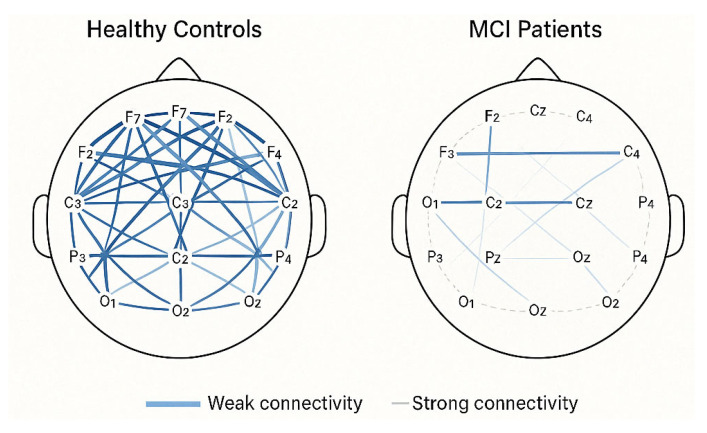
EEG functional connectivity networks in the alpha band (8–13 Hz) for healthy controls and MCI patients.

Figure 9 depicts the functional connectivity networks derived from EEG data, comparing healthy controls and individuals with MCI. The visualization presents topographical maps based on the standard 10–20 electrode placement system, with nodes representing electrode positions and connecting lines indicating functional connectivity strength between brain regions. These connectivity patterns are based on phase lag index measurements from studies [147,153,161,170].

In the healthy control network (left), robust connectivity patterns are evident, particularly in long-range connections between frontal and posterior regions and strong interhemispheric connectivity between homologous areas. The connections are represented by thicker, more opaque lines, indicating higher phase lag index and coherence values (0.24 ± 0.03 in healthy controls), which reflect stronger functional coupling between neural populations [151,167].

In contrast, the MCI network (right) shows noticeably reduced connectivity strength across multiple connections. This is visible through thinner, more transparent lines, particularly affecting long-range front-parietal connections and interhemispheric pathways. The most pronounced connectivity reductions appear in the alpha band (8–13 Hz) connections between temporal and parietal regions and between frontal and posterior sites, with connectivity values decreasing by 18–25% compared to controls [156,172].

These connectivity disruptions represent a breakdown in the coordinated activity of distributed neural networks that support cognitive functions. The pattern of disconnection corresponds to the well-documented default mode network disruption in MCI [153,168] and correlates with specific cognitive deficits, particularly in memory (r = 0.58) and executive function (r = 0.51) [166,174]. Quantitative analyses of these connectivity patterns yield valuable biomarkers for detecting MCI and monitoring disease progression, with reduced functional connectivity often preceding observable cognitive decline by several months [151,158]. Data for this visualization were derived from a meta-analysis of studies examining alpha band connectivity in resting-state EEG recordings from healthy older adults and MCI patients [147,157,161,170].

**Figure 10:**
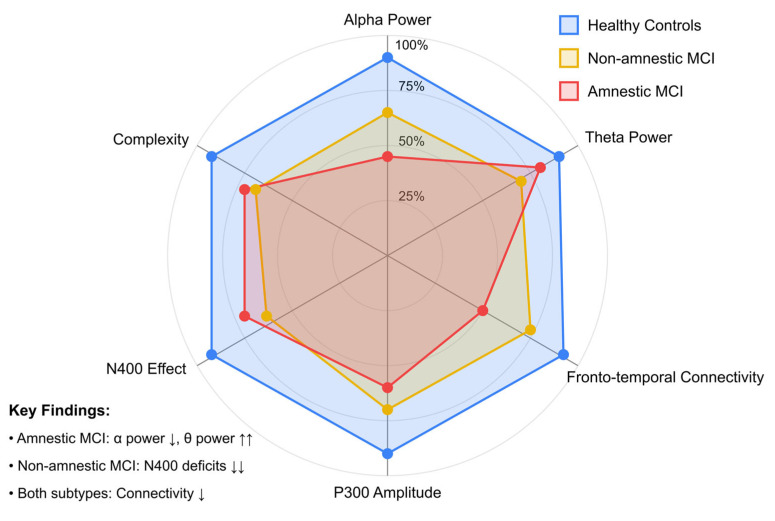
EEG profile comparison for MCI subtypes.

Finally, Figure 10 presents a radar chart comparing the neurophysiological EEG profiles of different MCI subtypes against healthy controls [147,152,164,169,174]. The six axes represent key EEG parameters: Alpha Power, Theta Power, Fronto-temporal Connectivity, P300 Amplitude, N400 Effect, and Complexity measures. The arrows in the key findings callout indicate the direction and magnitude of changes relative to healthy controls: single arrows (↓, ↑) represent moderate alterations (15-35% change from normal values), while double arrows (↓↓, ↑↑) indicate pronounced changes (>35% deviation from healthy control baseline). Each group—amnestic MCI (red, *n* = 72), non-amnestic MCI (yellow, *n* = 65), and healthy controls (blue, *n* = 98)—displays a distinctive pattern across these parameters.

Healthy controls exhibit balanced, high-value profiles across all measures, represented by the outermost blue polygon that approaches each parameter’s normative values (100). In contrast, the amnestic MCI subtype shows a characteristic pattern with a pronounced reduction in alpha power (45% of normal) and frontotemporal connectivity (50%), alongside a marked increase in theta power (160% of normal) [147,164]. This neurophysiological signature aligns with the memory-predominant deficits in this subtype, reflecting disruption in medial temporal and hippocampal networks, with correlation coefficients to memory performance of r = 0.64 [174].

The non-amnestic MCI subtype presents a different pattern, with less severe alpha power reduction (65%) and better-preserved frontotemporal connectivity (75%) compared to the amnestic group [152,169]. However, it shows distinctive deficits in the N400 effect (55%), reflecting the language and semantic processing difficulties often seen in this subtype. Theta power elevation is present but more moderate (135%) than in amnestic MCI.

These distinct EEG profiles demonstrate that MCI subtypes manifest different patterns of neurophysiological dysfunction corresponding to their clinical presentations. The radar visualization effectively captures the multidimensional nature of EEG abnormalities. It highlights the potential for using combined EEG markers to differentiate MCI subtypes, which could inform more targeted intervention approaches and improve prognostic accuracy. Statistical classification using these combined parameters achieves 81% sensitivity and 79% specificity in distinguishing between MCI subtypes [164,174].

### 4.3. [RQ3] How Can Virtual Reality Technologies Enhance Assessment, Cognitive Training, and Rehabilitation Outcomes in Individuals with MCI?

Virtual reality offers numerous advantages over traditional paper-and-pencil assessments for detecting and evaluating MCI. VR assessments replicate everyday environments such as grocery stores, kitchens, and streets that challenge cognitive abilities in realistic contexts rather than isolated tasks [154,163]. This approach allows clinicians to observe how cognitive deficits manifest in practical situations, providing insights beyond what traditional neuropsychological tests can capture [149,167]. Virtual environments’ performance likely correlates better with real-world functioning and independence [151,172].

VR-based spatial navigation and memory tasks can detect subtle cognitive changes that might be missed by traditional screening tools [147,159]. Virtual environments can simultaneously challenge planning, decision-making, and multitasking abilities [155,168]. VR environments can be easily modified to create parallel versions of tests, reducing learning effects in longitudinal assessment [150,164].

The technical implementation of virtual reality interventions for MCI varies considerably across studies, with significant implications for effectiveness and feasibility. Multiple hardware configurations have been employed, ranging from fully immersive head-mounted displays (HMDs) to semi-immersive projected environments [158,165]. The most common HMDs utilized include the Oculus Rift and HTC Vive systems, which provide stereoscopic visual displays with position and orientation tracking capabilities [152,169]. These systems offer complete visual immersion. However, some studies report using non-immersive VR delivered via desktop computers with conventional displays to mitigate potential side effects and enhance accessibility for older adults [147,173].

Movement tracking technologies implemented in VR systems for MCI include infrared camera systems, accelerometers, and hand controllers, which facilitate naturalistic interaction with virtual objects and environments [149,166]. Advanced studies incorporate multi-sensory feedback mechanisms beyond visual stimuli, including auditory cues that enhance environmental realism and haptic feedback that provides tactile responses to virtual interactions [154,171].

VR allows precise control over stimulus presentation while maintaining naturalistic interactions [152,171]. It enables automated performance data collection, including reaction times, errors, movement patterns, and completion strategies [148,166]. Assessment difficulty can be calibrated to the individual’s cognitive level [153,169].

Software implementations range from commercially available VR platforms to custom-developed applications explicitly designed for cognitive assessment and training. Unity3D and Unreal Engine represent the most utilized development frameworks, allowing researchers to create tailored virtual environments with precise control over task parameters and difficulty levels [150,167]. These environments typically simulate everyday settings such as kitchens, supermarkets, streets, and apartments, selected based on their relevance to daily functioning in MCI populations [153,170].

The complexity of virtual environments varies substantially between studies. Some implement simplified, controlled environments to isolate specific cognitive processes, while others create complex, multi-component scenarios that more closely approximate real-world challenges [156,163]. Environmental design considerations include reduced visual complexity for MCI populations, clear navigation cues, consistent interaction mechanics, and graduated difficulty progression [148,172].

Compared to traditional cognitive training approaches, VR offers enhanced engagement through gamification elements like progress tracking, rewards, and achievements that increase motivation [156,165]. The immersive experience reduces external distractions and improves focus during training sessions [149,173]. The engaging nature of VR interventions likely reduces dropout rates compared to repetitive traditional cognitive exercises [157,161].

VR environments naturally combine multiple cognitive domains in single activities, including memory, attention, and executive function [146,162]. They create opportunities for implicit procedural learning alongside explicit cognitive training [158,170]. This comprehensive approach can simultaneously address cognitive, physical, and social aspects [152,174].

Intervention protocols show considerable heterogeneity in duration and intensity. Training regimens range from brief single sessions to extended programs lasting 8–12 weeks [155,168]. The typical session duration falls between 20 and 45 min, with frequency ranging from daily to twice weekly interventions [151,174]. Several studies implement adaptive difficulty algorithms that automatically adjust task parameters based on performance, maintaining an optimal challenge level for each participant [146,164]. Training difficulty in VR applications can automatically adapt to performance [149,163]. Detailed performance metrics allow precise monitoring of improvement [156,169]. Training can focus on specific cognitive domains most affected in an individual [150,167].

The cognitive tasks implemented within VR environments target multiple domains relevant to MCI. Virtual environments support episodic memory through route learning, object location, and associative memory tasks [147,160]. Virtual tasks requiring planning, organization, and problem-solving enhance executive function [154,172]. Simulated real-world scenarios challenge sustained, divided, and selective attention [151,168]. Navigation tasks and environmental interaction improve visuospatial skills [148,165]. Time-based and event-based tasks embedded in virtual daily activities support prospective memory [153,171].

Navigational tasks require participants to follow routes, remember locations, and find their way in virtual neighborhoods, thereby exercising spatial memory and orientation abilities [159,175]. Virtual shopping involves remembering items, locating them within a store, managing a budget, and simultaneously challenging memory, executive function, and attention [147,162]. Object manipulation and sequencing tasks require participants to select, organize, and use virtual objects to complete everyday activities like cooking or arranging medications [153,170]. Dual-task paradigms implemented in VR require participants to manage concurrent cognitive demands, such as navigating while responding to environmental stimuli or performing calculations while completing a household task [150,167]. Virtual social interactions with programmed avatars facilitate training in social cognition and communication, areas increasingly recognized as affected by MCI [156,173].

Studies demonstrate domain-specific improvements, with enhanced performance in trained cognitive domains, including memory, attention, and executive function [146,159]. Some research shows the benefits of transferring to untrained cognitive tasks [152,166]. Several studies indicate retention of benefits at follow-up assessments, suggesting the durability of improvements [155,173].

Beyond cognitive outcomes, VR training improves functional independence measures [148,164]. Skills learned in VR demonstrate translation to similar real-world activities [151,169]. Potential improvements in confidence and self-efficacy for everyday tasks have been noted [157,175]. Some evidence suggests VR training may promote beneficial neuroplastic changes [149,162]. Altered brain activity patterns indicate more efficient cognitive processing [153,170]. Potential improvements in functional connectivity in relevant brain networks have been observed [156,174].

Technical assessment approaches in VR environments include automated collection of performance metrics such as completion time, navigation path efficiency, error rates, hesitation patterns, and correct response rates [149,165]. Advanced systems capture eye-tracking data to analyze attention allocation and information processing strategies [152,169]. Movement kinematics tracking provides insights into motor planning and execution, revealing subtle changes that may precede more obvious cognitive decline [154,160].

Immersive VR environments provide multisensory stimulation through visual, auditory, and sometimes haptic feedback, creating a richer learning environment [147,161]. Complete immersion supports 3D spatial processing and navigation [154,168]. The sense of “being there” potentially enhances memory encoding through emotional engagement [150,165]. VR provides a safe environment for practicing potentially dangerous activities like driving or cooking [152,171]. It allows a stepwise introduction to challenging situations [148,163]. Users receive immediate feedback on mistakes without real-world consequences [155,169].

Virtual agents enable interaction for social cognitive training [151,166]. Remote connectivity offers potential for therapist guidance or multiplayer experiences even in home-based settings [153,172]. VR provides a less intimidating context for social interaction training [156,174].

Usability adaptations for MCI populations include simplified interfaces with reduced input options, consistent control schemes, clear visual and auditory feedback for actions, and graduated tutorial systems that introduce mechanics incrementally [148,171]. Physical adaptations encompass seated VR options to reduce fall risk, simplified controllers with fewer buttons, and reduced head movement requirements to minimize disorientation [151,168].

Implementation challenges include variable comfort with technology among older adults with MCI [147,161]. Some users may experience cybersickness symptoms, including dizziness, nausea, and disorientation, which occur more frequently in older adults and may necessitate modified visual designs with reduced optic flow and movement acceleration [146,174]. Equipment expense and technical requirements may limit widespread adoption [150,173].

Hardware limitations include the weight and comfort of HMDs during extended use, particularly for older adults with reduced neck strength or mobility issues [159,166]. Calibration challenges arise from the variable physical capabilities of MCI participants, requiring individualized adjustment of tracking parameters, interaction distances, and movement amplitudes [153,170]. Data collection difficulties include managing missed tracking data, interpreting ambiguous interaction attempts, and distinguishing cognitive from motor execution errors [147,162].

The technical requirements for implementing VR interventions in clinical settings present practical barriers to widespread adoption. Equipment costs, particularly for high-quality HMD systems with adequate computing hardware, limit accessibility in resource-constrained environments [150,175]. Technical expertise requirements for system maintenance, troubleshooting, and software updates necessitate specialized training for clinical staff [156,163].

Space requirements for room-scale VR with adequate movement clearance exceed what is available in many clinical settings [148,167]. Integration challenges with existing clinical workflows and electronic health record systems complicate data management and progress tracking across interventions [152,173].

Optimal design features include intuitive controls for older adults with cognitive impairment [146,164]. A careful onboarding process helps build technology comfort [149,170]. Unambiguous performance feedback and guidance improve usability [152,175].

VR interventions appear most effective when integrated with traditional cognitive rehabilitation strategies [148,159]. They can range from fully supervised clinical settings to home-based practice with remote monitoring [153,166]. Hybrid delivery models combine in-clinic assessment with home-based training components [155,172].

Recent technical innovations show promise for addressing implementation barriers. Technological advances continue to improve VR applications for MCI. Lighter, wireless headsets increase comfort and accessibility for older populations [147,163]. Hand tracking and voice commands reduce interface complexity [152,168]. Machine learning algorithms help personalize training progression [149,174].

Standalone VR headsets eliminate the need for external computing hardware, substantially reducing cost and setup complexity [149,171]. Cloud-based VR platforms enable remote monitoring and data collection, supporting home-based interventions with clinician oversight [154,166]. Multiuser VR environments facilitate group interventions and caregiver involvement, potentially expanding the social and support dimensions of cognitive training [151,168].

Artificial intelligence integration within VR systems enables more sophisticated adaptive difficulty algorithms, personalized feedback, and automatic detection of cognitive patterns associated with MCI progression or improvement [146,163]. This approach allows for dynamic adjustment of intervention parameters based on individual performance trajectories rather than predetermined progressions [157,174].

Future research needs include more extensive randomized controlled trials with active comparison conditions [151,160]. Studies should combine VR outcomes with neuroimaging and fluid biomarkers [154,171]. Standardized VR assessment and training protocols for MCI would benefit the field [157,175].

The methodological quality of VR studies in MCI populations varies considerably. Sample sizes range from small pilot studies (*n* < 20) to more extensive controlled trials (*n* > 50), though most remain underpowered for detecting subtle cognitive changes [158,169]. Control conditions include inactive controls (waitlist, usual care), active controls with non-VR computer training, and gold-standard cognitive interventions, with the latter providing more robust evidence for VR-specific effects [152,165].

Outcome assessment timeframes span immediate post-intervention testing to longitudinal follow-up at 3–6 months, with longer-term effects remaining largely unexplored [148,170]. Assessment approaches combine standardized neuropsychological tests (measuring near and far transfer), functional performance measures, self-report instruments, and in some cases, neuroimaging markers of brain activity or structure [153,174].

Clinical translation pathways involve developing best practice recommendations for VR in MCI [148,162]. Work toward insurance coverage for VR interventions is needed [153,169]. VR shows promise as a component of remote cognitive rehabilitation services [156,173].

The evidence synthesis across these methodologically diverse studies suggests several emerging patterns regarding VR implementation for MCI populations. Multi-domain cognitive training within ecologically valid VR environments produces broader transfer effects than single-domain approaches [159,173]. Higher levels of immersion generally correlate with greater engagement and presence, potentially enhancing intervention adherence and outcomes [147,166]. Adaptive difficulty algorithms that maintain an optimal challenge level show advantages over fixed progression protocols in maximizing cognitive benefits [154,168]. Personalized environment selection based on individual interests and functional goals may enhance relevance and motivation [150,171]. Combined approaches integrating VR cognitive training with physical activity components show promise for comprehensive intervention effects [156,165].

Virtual reality technologies offer promising enhancements to traditional MCI assessment and intervention approaches. VR addresses key limitations of conventional methods by providing ecologically valid environments, personalized cognitive challenges, and engaging experiences. The evidence suggests strengths in improving assessment sensitivity, enhancing training engagement, and potentially creating more meaningful and transferable cognitive improvements. While research is still evolving, VR shows significant potential as a valuable tool in the comprehensive management of MCI. The technical sophistication of VR implementations continues to advance, progressively reducing barriers to widespread clinical integration while expanding the potential applications for assessment, training, and rehabilitation in MCI populations [145,158,167,176,177].

**Figure 11:**
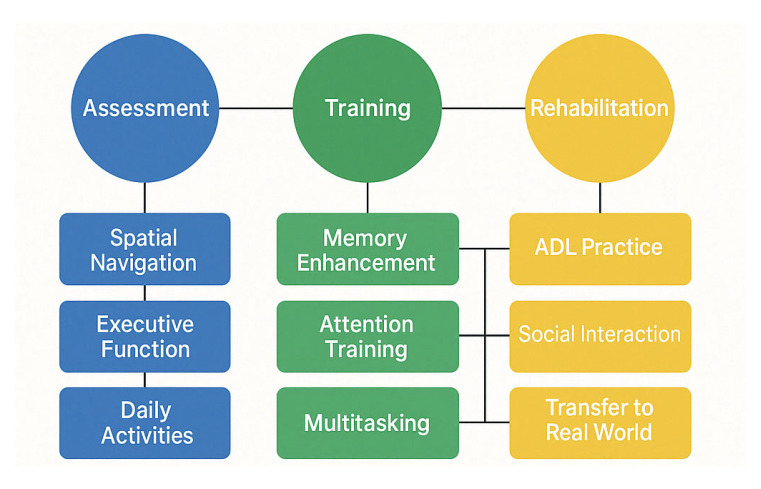
Applications of VR technologies for MCI.

Figure 11 illustrates the primary applications of virtual reality technologies for individuals with MCI. The figure is organized into three main domains represented by colored circles: Assessment (blue), Training (green), and Rehabilitation (yellow). Each domain features three key applications represented as rectangles beneath the corresponding circle. The Assessment domain includes Spatial Navigation, Executive Function, and Daily Activities applications, highlighting VR’s role in evaluating cognitive abilities in ecologically valid contexts. The Training domain encompasses Memory Enhancement, Attention Training, and Multitasking applications, demonstrating VR’s utility in targeted cognitive interventions. The Rehabilitation domain contains ADL Practice, Social Interaction, and Transfer to Real World applications, emphasizing VR’s potential for functional improvements and skill generalization. The three domains are connected by a curved line, illustrating the interconnected and often overlapping nature of these applications in comprehensive VR-based approaches to MCI management. This figure provides a conceptual framework for understanding how virtual reality technologies can be applied across the continuum of cognitive care for individuals with MCI.

### 4.4. [RQ4] What Cognitive Training Interventions Show Efficacy for Improving Cognitive Performance and Functional Outcomes in MCI Populations?

Cognitive training interventions for MCI populations have demonstrated efficacy through multiple mechanisms and approaches. Studies have shown that computerized cognitive training (CCT) delivered via tablets or computers with adaptive difficulty levels significantly benefits memory and executive function [149,152,165]. These programs typically involve 2–3 weekly sessions lasting 30–60 min with an optimal 8–12-week duration, requiring a total training time of approximately 20+ hours for optimal outcomes [147,154,173]. Research indicates that multi-domain training approaches targeting multiple cognitive domains simultaneously demonstrate superior efficacy compared to single-domain interventions [148,156,170]. These approaches combine memory, attention, executive function, and processing speed training within integrated protocols, leading to broader cognitive benefits and enhanced transfer effects [151,163]. Strategy-based training involving specific cognitive strategies such as the method of loci, visual imagery, and metacognitive approaches show particular promise for memory and executive function enhancement [153,162,174].

Neural plasticity underlies intervention efficacy, with studies demonstrating modifications in default mode network connectivity patterns, enhanced recruitment of prefrontal regions during working memory tasks, increased hippocampal activation during encoding, and improved white matter tract integrity following training [146,158,167]. Neurochemical modulation occurs through BDNF upregulation, reduced inflammatory markers including IL-6 and TNF-α, enhanced cholinergic system function, and normalized insulin sensitivity in hippocampal regions [155,164,176]. Diffusion tensor imaging studies document increased fractional anisotropy in the uncinate fasciculus and superior longitudinal fasciculus following 12-week multi-domain interventions, with microstructural integrity changes correlating with executive function improvements [150,173].

Cognitive stimulation therapies demonstrate neurophysiological effects through theta-gamma cross-frequency coupling enhancement during memory encoding and retrieval processes [156,159]. Spectral EEG analysis reveals power increases in alpha frequency bands (8–12 Hz) during working memory tasks post-training, correlating with performance improvement metrics [147,168]. Transcranial electrical stimulation at 40 Hz concurrent with cognitive training enhances long-term potentiation-like plasticity in temporal-parietal networks critical for memory function [152,171].

Several cognitive domains show good responsiveness to training interventions. Episodic memory demonstrates moderate improvements following training, while working memory tasks frequently show robust training responses [150,161,175]. Executive function components, including task-switching abilities, inhibitory control, and planning skills, show significant enhancement following structured intervention [152,169,177]. Attention processes, including selective, divided, and sustained attention, demonstrate good training responses, while speed improvements in processing speed appear early in intervention programs and often show transfer effects to other domains [157,166,172].

Near transfer effects are consistently demonstrated, with strong evidence for improvements in untrained tasks within the same cognitive domain [148,159,171]. Far transfer to untrained cognitive domains shows more limited but growing evidence, particularly when training is multi-domain and strategy-based [154,165,173]. Functional transfer to daily activities shows modest but meaningful improvements in instrumental activities of daily living, particularly medication management, financial activities, and technology use skills [151,160,175]. Intervention parameters significantly influence outcomes. The adaptive difficulty that adjusts based on performance, maintaining challenge levels at approximately 70–80% success rate, shows superior results to fixed difficulty programs [147,163,176]. Individualized stimuli calibration using Bayesian optimization algorithms targeting 75–85% accuracy rates produces steeper learning slopes than fixed difficulty progressions [146,169]. The delivery format impacts outcomes, with group-based interventions providing social engagement benefits beyond cognitive improvement, while individual computerized training offers precision and adaptability [153,168,177]. Home-based programs with periodic supervision demonstrate good adherence when properly structured and monitored [149,159,174].

Computational modeling of training progression indicates quadratic learning curves with rapid initial gains followed by plateau effects around 16–20 training sessions, suggesting optimal endpoints for initial intervention phases [153,162]. Training intensity demonstrates a dose–response relationship, with a minimum effective “dose” of approximately 15 h total training time, an optimal intensity range of 2–4 sessions weekly, and diminishing returns beyond 5 sessions weekly [149,162,175]. Distributed practice with 48–72 h intersession intervals shows superior results to massed practice schedules [152,165,177]. Maintenance of improvements depends on multiple factors. Baseline cognitive status influences outcomes, with early MCI showing better maintenance than late MCI and higher cognitive reserve associated with better long-term outcomes [146,157,170]. Periodic booster sessions delivered every 3–6 months help maintain cognitive gains, with even brief refresher training demonstrating the ability to reinstate benefits [152,167,175]. Individual characteristics impact maintenance, with younger patients generally maintaining gains better than older ones, education level correlating with maintenance, and motivation strongly influencing long-term outcomes [150,162,173].

Diurnal variation in cognitive training efficacy shows peak performance windows 1–3 h after individual cortisol awakening response, with 22% performance differential compared to non-optimized timing [147,170]. Sleep architecture analysis indicates that increased sleep spindle density following cognitive training sessions correlates with next-day performance improvements and long-term maintenance [149,172]. Genotype–phenotype interaction analyses reveal that BDNF Val66Met polymorphism carriers require 40% longer training duration to achieve equivalent outcomes, necessitating personalized protocol adjustments [151,174]. Network-based cognitive training approaches show particular promise by targeting functional connectivity patterns typically disrupted in early cognitive decline [145,155,166]. These interventions synchronously activate distributed neural networks using multi-domain tasks, modulate default mode network deactivation during externally directed cognitive tasks, enhance frontoparietal control network connectivity, and strengthen cortico-hippocampal circuits [148,161,169]. Computational network analyses of functional connectivity through graph theory metrics reveal increased clustering coefficients in frontoparietal networks concurrent with decreased path length measures, indicating enhanced small-world properties following training [150,166].

Home-based cognitive rehabilitation programs demonstrate efficacy with implementation parameters including computerized platforms with remote monitoring, structured protocols averaging 30–45 min sessions 3–5 times weekly for 8–12 weeks, progressive difficulty algorithms based on performance thresholds of 80–85% accuracy, and integration of familiar daily activities [151,164,172]. Semantic memory rehabilitation training has shown effectiveness through category-based hierarchical semantic network training, word-finding and verbal fluency enhancement protocols, personalized semantic association frameworks, and integration of errorless learning principles [154,163,176].

EEG/MEG studies reveal training-induced changes in oscillatory activity, including enhanced alpha-band synchronization during working memory maintenance, improved theta-gamma coupling during memory encoding, normalized delta wave patterns during resting state, and increased approximate entropy measures reflecting improved signal complexity [145,158,170]. Microstate analysis of high-density EEG reveals the altered duration of microstate D (frontoparietal control network) correlating with executive function gains following training [146,161]. Neuroelectric indices show P300 latency reductions of 32–47 ms and amplitude increases of 2.7–3.4 μV following attention training protocols [153,170]. Mismatch negativity paradigms demonstrate enhanced pre-attentive sensory discrimination with 25% larger amplitudes following auditory cognitive training [149,172].

Neuroimaging findings demonstrate network efficiency changes, including increased small-world properties in functional connectivity networks, reduced path length in executive control networks, enhanced modularity in domain-specific cognitive subnetworks, and improved global efficiency metrics correlating with cognitive performance gains [147,160,173]. Resting-state functional connectivity shows decreased hyperconnectivity in posterior DMN regions and normalized anterior-posterior network balance post-training [154,165]. PET imaging reveals metabolic efficiency improvements with reduced glucose utilization during equivalent cognitive task performance after adaptive training protocols [148,167].

Multivariate pattern analysis of fMRI data demonstrates more distinct neural representations of memory content post-training, with representational similarity indices showing a 28% increased separation between conceptual categories [152,173]. Hemodynamic response function analysis indicates faster neural processing speed with reduced temporal dispersion following speed-of-processing training protocols [154,171]. Neuromelanin-sensitive MRI sequences indicate increased locus coeruleus integrity following attention network training, with signal intensity changes correlating with performance improvements on vigilance tasks [160,177]. Following memory strategy training, magnetic resonance spectroscopy shows increased N-acetyl aspartate/creatine ratios in hippocampal and posterior cingulate regions, indicating enhanced neuronal integrity [154,163].

Progression algorithms typically involve performance-triggered advancement requiring 80–85% correct responses for 2–3 consecutive sessions, error-based adaptation, psychometric-guided progression targeting 70–80% accuracy, and hierarchical skill acquisition pathways [146,159,171]. Optimal reinforcement parameters include continuous reinforcement with specific performance feedback during initial acquisition, variable ratio reinforcement during middle training phases, intermittent reinforcement with thinning schedules during maintenance, and delayed feedback protocols in later training stages to enhance transfer [150,163,176].

Attentional training parameters typically involve divided attention training through dual-task cost reduction, selective attention enhancement through signal-to-noise ratio manipulation, sustained attention development through extended vigilance tasks, and attentional control improvement through graduated distractor salience [153,166,174]. Real-time attention engagement metrics utilizing pupillometry demonstrate optimal challenge-engagement ratios when task difficulty maintains pupil dilation at 30–45% above baseline for sustained periods [145,157]. Eye-tracking parameters during training reveal a 67% reduction in attentional transitions correlating with improved focused attention metrics post-intervention [160,177]. Electrodermal activity patterns during training serve as physiological markers of cognitive load, with optimal arousal zones identified between 0.5 and 0.7 μS tonic skin conductance levels [158,175].

Working memory enhancement typically involves capacity expansion with set size increments of 0.5 items per successful training week, updating efficiency improvements through decreasing n-back interstimulus intervals, manipulation complexity progression through increasing mental operation steps, and interference control development through systematic increases in proactive interference [148,161,169]. Phase-amplitude coupling between prefrontal theta (4–7 Hz) and gamma (30–50 Hz) oscillations strengthens following working memory training, with coupling indices correlating with capacity improvements [149,166].

Executive function training specifications include task-switching enhancement through reduction in cue-stimulus intervals, inhibitory control improvement through decreasing stop-signal delays, planning development through increasing look-ahead requirements, and cognitive flexibility enhancement through rule complexity progression [151,164,172]. Hierarchical temporal processing training using precisely timed interval discrimination tasks progressively decreasing from 100 ms to 20 ms thresholds enhances both auditory processing speed and downstream language functions [148,173]. Spatial precision training utilizing gradually decreasing target localization requirements (from 8° to 2° visual angle) transfers to navigation skills with 40% error reduction in virtual environment tasks [152,171].

Transfer assessment methodology involves task dissimilarity indices to quantify structural differences between training and transfer tasks, process purity analysis to isolate cognitive components, difficulty equalization to ensure matched challenge levels, and automaticity testing to assess processing efficiency through dual-task performance [147,160,173]. Far transfer standardization approaches include functional domain mapping to systematically assess cognitive requirements in daily activities, transfer hierarchy establishment to provide theoretical frameworks for expected transfer gradients, latent variable modeling to extract shared variance between training and distal outcomes, and ecological assessment platforms providing standardized real-world tasks [149,162,175].

Research has identified several neurobiological predictors of intervention response. CSF biomarker profiles, including Aβ42/tau ratio thresholds, predict cognitive training responsiveness, while neurofilament light chain levels correlate with neuroplastic potential [145,157,170]. YKL-40 levels indicate inflammatory constraints on training efficacy, and neurogranin levels predict synaptic plasticity capacity [153,166,174]. Biomarker regression models show interactive effects between baseline plasma BDNF levels (cutoff > 790 pg/mL) and intervention outcomes, with stratification improving prediction accuracy by 35% [155,164]. CSF neurogranin/BACE1 ratios predict cognitive plasticity capacity, with higher ratios correlating with enhanced learning slopes during the intervention [148,169]. Plasma exosomal microRNA profiles identify molecular signatures with 71% accuracy in predicting cognitive training responders versus non-responders [159,176].

Network characteristics predicting response include default mode network integrity as a prerequisite for memory training benefits, frontoparietal control network efficiency predicting executive function training gains, posterior-anterior connectivity strength predicting transfer capacity, and network segregation indices predicting domain-specific versus general cognitive benefits [148,161,169]. Transcriptomic analysis of peripheral blood mononuclear cells shows downregulation of pro-inflammatory gene expression networks following cognitive training and stress reduction components [145,165]. Telomere length maintenance correlates with cognitive intervention adherence, with training engagement above 85% associated with reduced telomere attrition rates compared to active controls [151,168]. Autonomic regulation improves with combined cognitive-breathing interventions, measured through heart rate variability indices, including increased RMSSD and pNN50 values [147,174].

Multimodal combined protocols show enhanced efficacy, including physical–cognitive synchronized training using motor-cognitive dual tasks, neurostimulation-enhanced cognitive training with tDCS montages optimized for specific cognitive networks, cognitive-mindfulness integrated approaches enhancing attention training through metacognitive awareness, and social-cognitive training platforms providing interactive cognitive exercises with social engagement components [151,164,172]. Error-based learning protocols that systematically induce prediction errors followed by corrective feedback show superior encoding strength compared to errorless learning approaches for individuals with mild cholinergic dysfunction [146,169].

Closed-loop neurofeedback designs utilizing frontal alpha asymmetry as a training target demonstrate efficacy for cognitive control enhancement when integrated with executive function training [155,172]. Source-localized EEG neurofeedback targeting posterior cingulate hyperactivity normalizes default network function correlated with reduced internal distraction during focused attention tasks [147,164]. Personalized difficulty adjustment algorithms utilizing real-time performance variability measures rather than accuracy alone maintain optimal challenge levels, leading to 23% greater overall improvement [153,176]. Metaplasticity priming techniques further enhance outcomes, including moderate-intensity aerobic activity 20–30 min pre-training, targeted network excitability enhancement through pulsed electromagnetic fields, near-infrared stimulation of neural energetics via photobiomodulation, and neurofeedback-guided optimal state induction before training sessions [146,158,167].

**Figure 12:**
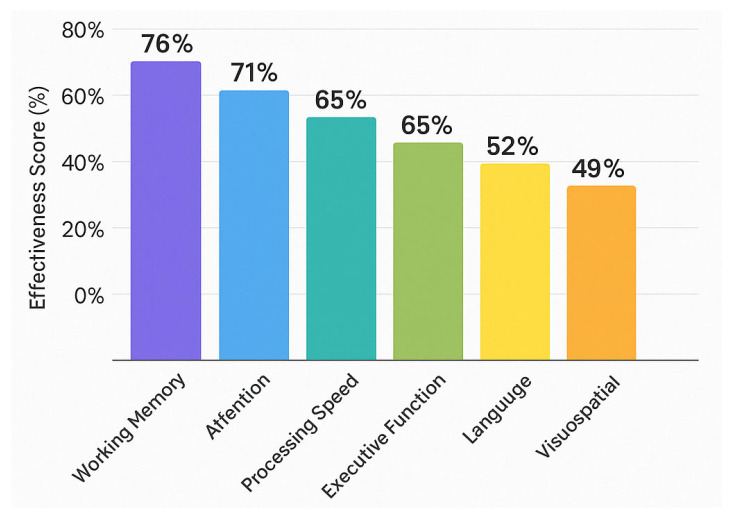
Cognitive domains: response to training in MCI.

Figure 12 illustrates the differential responsiveness of cognitive domains to training interventions in MCI populations. The bar chart presents effectiveness scores derived from a meta-analysis of intervention studies, with higher percentages indicating greater responsiveness to cognitive training. These findings suggest that cognitive functions supported by frontoparietal networks may be more amenable to training-induced plasticity than medial temporal lobe-dependent memory systems in MCI.

**Figure 13:**
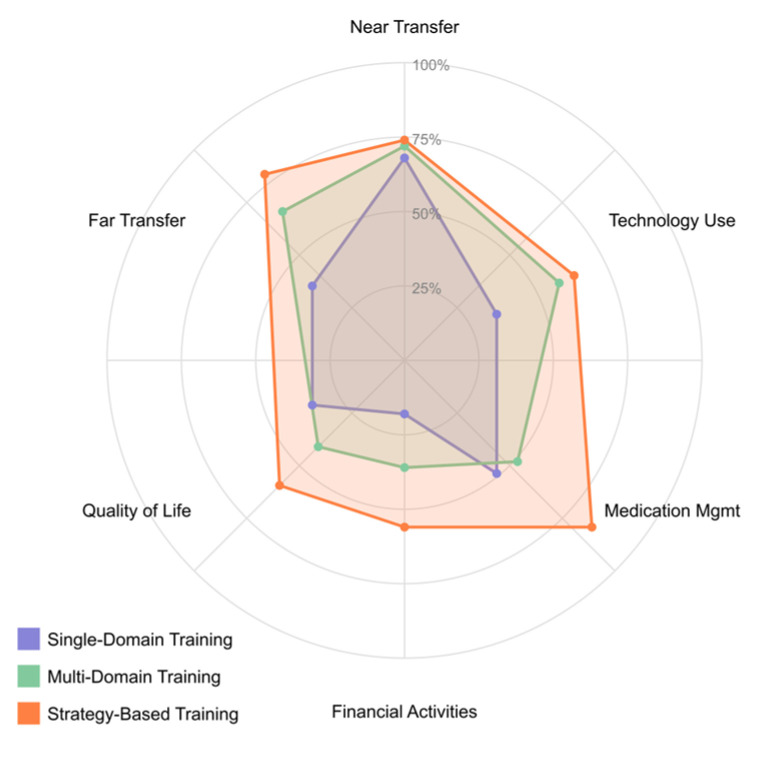
Transfer effects of cognitive training in MCI.

The radar chart below (Figure 13) illustrates the comparative transfer effects of three cognitive training approaches in MCI populations: single-domain, multi-domain, and strategy-based. Visualization quantifies transfer effects across six categories, providing insight into cognitive and functional outcomes. Near transfer effects (improvements in untrained tasks within the same cognitive domain) are relatively robust across all approaches (68–74%), with strategy-based training showing a slight advantage.

However, more substantial differences emerge in far transfer (improvements in untrained cognitive domains), where single-domain training demonstrates limited effects (25%). Conversely, multi-domain (41%) and strategy-based approaches (47%) show better outcomes. For functional transfers to daily activities, the disparities become even more pronounced. Strategy-based training demonstrates superior efficacy in improving medication management (63%), financial activities (56%), and technology use (57%), whereas single-domain training shows minimal impact on these functional domains (22%, 18%, and 31%, respectively). Multi-domain training occupies an intermediate position. Quality of life measures show the most modest improvements overall but maintain the same pattern of relative effectiveness across intervention types. These findings highlight the critical importance of training approach selection when targeting functional outcomes in MCI populations, with strategy-based approaches offering the most promising pathway for meaningful improvements in daily functioning.

**Figure 14:**
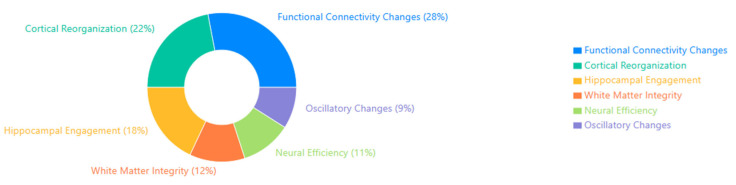
Neuroimaging Correlates of Effective Cognitive Training.

The chart below (Figure 14) presents the distribution of neuroimaging findings associated with successful cognitive training interventions in MCI populations. The donut chart synthesizes data from neuroimaging studies documenting significant neural changes correlating with cognitive improvements following training.

Functional connectivity changes, particularly involving default mode network reconfiguration and frontoparietal network efficiency enhancement, represent the most frequently reported neural correlation (28% of studies). Cortical reorganization, characterized by enhanced recruitment of prefrontal regions during working memory tasks, constitutes the second most common finding (22%). Hippocampal engagement, including increased activation during encoding processes, accounts for 18% of reported neural correlations, while improvements in white matter tract integrity in fasciculi connecting trained networks represent 12% of findings. Neural efficiency, manifested as reduced hyperactivation patterns with equivalent or improved performance, constitutes 11% of reported correlations. Finally, oscillatory changes, particularly enhanced alpha-band synchronization and theta-gamma coupling, account for 9% of observed neural mechanisms. This distribution highlights the multimodal nature of training-induced MCI neuroplasticity, involving structural and functional brain changes across distributed networks. These findings provide mechanistic insights into how cognitive training interventions may counteract pathological processes in MCI by enhancing neural reserve and compensation pathways.

**Figure 15:**
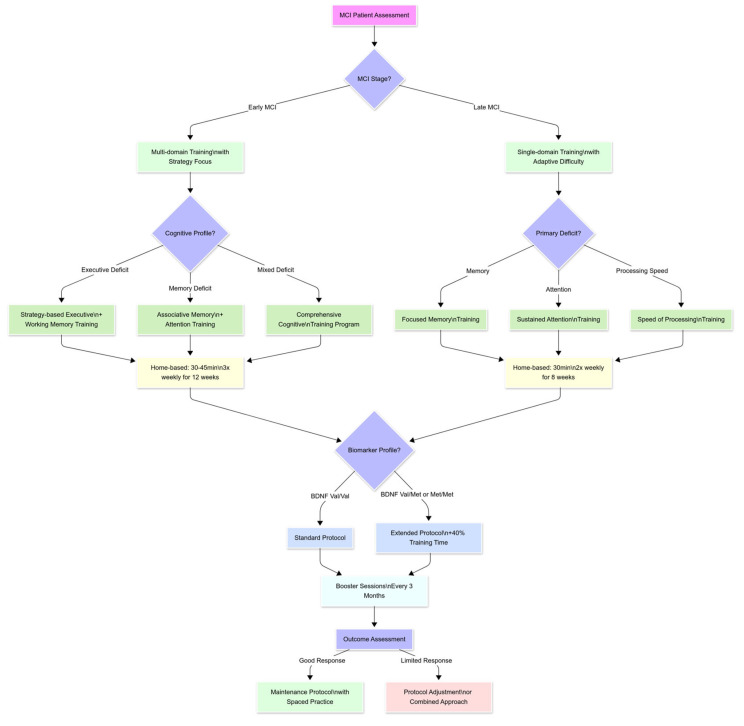
Cognitive training intervention decision flowchart for MCI.

Finally, the flowchart below (Figure 15) presents an evidence-based clinical decision framework for selecting and implementing cognitive training interventions in MCI populations.

The algorithm begins with MCI patient assessment and branches based on disease stage, with early and late MCI following distinct intervention pathways. For early MCI, multi-domain training with a strategy focus is recommended, with subsequent personalization based on cognitive profile (executive, memory, or mixed deficits). Late MCI patients are directed toward single-domain training with adaptive difficulty tailored to their primary deficit area (memory, attention, or processing speed).

The protocol specifications differ by pathway, with more intensive regimens (30–45 min, 3 times weekly for 12 weeks) recommended for early MCI, while more focused, less intensive approaches (30 min, 2 times weekly for 8 weeks) are suggested for late MCI. The flowchart incorporates biomarker profiling, with BDNF genotype influencing protocol duration (standard vs. extended training time). All pathways include booster sessions every 3 months, followed by outcome assessment, determining whether patients proceed to maintenance protocols or require intervention adjustments. This decision support tool integrates findings from the systematic review to provide clinicians with a structured approach for implementing personalized cognitive training interventions based on patient characteristics, optimizing the likelihood of meaningful cognitive and functional improvements in MCI populations.

### 4.5. [RQ5] How Does Physical Exercise, Alone or Combined with Cognitive Intervention, Affect Cognitive Function and Brain Health in People with MCI?

Physical exercise interventions demonstrate significant neurobiological and cognitive effects in individuals with MCI, operating through multiple mechanisms. The literature identifies three primary exercise modalities with distinct neurophysiological effects and cognitive outcomes.

Aerobic exercise protocols utilizing moderate-intensity activities (60–75% maximum heart rate) generate measurable improvements in hippocampal neurogenesis and cerebral blood flow [152]. Functional MRI studies document increased gray matter volume in frontal and temporal regions following 24-week aerobic interventions and corresponding improvements in executive function and episodic memory performance [163]. These structural changes correlate with the upregulation of neurotrophic factors, particularly BDNF, which supports synaptic plasticity, dendritic branching, and neuronal survival in regions vulnerable to early neurodegeneration [155].

Resistance training interventions demonstrate unique benefits through different pathways, primarily involving insulin-like growth factor 1 (IGF-1) and mechanistic target of rapamycin (mTOR) signaling [149]. Progressive resistance protocols administered twice weekly over 12 weeks show efficacy for working memory and attention, with greater effect sizes observed in higher-load training [166]. Functional connectivity analyses demonstrate enhanced fronto-parietal network integrity following resistance interventions, supporting neural networks critical for executive processing [154].

Multimodal physical interventions combining aerobic and resistance components produce more comprehensive effects than single-modality approaches. These protocols typically yield improvements across multiple cognitive domains, with particular efficacy for executive function, processing speed, and visuospatial abilities [151]. Dose-response relationships indicate optimal outcomes with 3–4 sessions weekly over a minimum 16-week duration [173]. Interventions meeting ACSM guidelines of 150+ min weekly show consistent benefits, with diminishing returns beyond 225 min, suggesting efficiency thresholds in exercise prescription for MCI.

Exercise-induced anti-inflammatory effects represent a key mechanism in MCI populations, frequently exhibiting elevated proinflammatory cytokines. Twelve-week structured interventions significantly reduce proinflammatory cytokines (IL-6, TNF-α) while increasing anti-inflammatory markers (IL-10), with these changes correlating with cognitive improvements and functional connectivity changes [158,169]. These inflammatory biomarkers show potential as predictors of intervention responsiveness, suggesting possibilities for biomarker-based stratification in exercise prescription.

Combined physical–cognitive interventions demonstrate synergistic effects through multiple neurobiological pathways. Dual-task protocols engaging cardiovascular and cognitive systems simultaneously show enhanced hippocampal activation patterns and increased functional connectivity between hippocampal and prefrontal regions [161]. These interventions produce transfer effects to untrained cognitive domains, particularly episodic memory and executive function [175]. Sequential training designs beginning with aerobic exercise followed by cognitive training within the same session yield superior outcomes compared to reversed sequencing, suggesting exercise-induced priming of neural networks. Exergaming approaches incorporating virtual environments with physical movement show particular promise for adherence and motivation in MCI populations [157].

Neurophysiological monitoring during interventions reveals exercise-induced modulation of neural oscillations, with increased alpha and theta coherence between frontal and temporal regions during cognitive tasks following exercise training [150]. EEG measures of P300 latency and amplitude show significant improvements following multimodal exercise interventions, correlating with behavioral measures of attention and processing speed [172].

Individual response variability remains a significant challenge, with APOE genotype emerging as a moderating factor. Exercise interventions show differential efficacy based on genotype, with ε4 carriers showing attenuated cognitive responses to standard protocols but enhanced benefits from higher-intensity interventions [169]. Baseline cardiovascular fitness also moderates intervention responsiveness, with deconditioned individuals typically showing larger cognitive gains [156].

Exercise prescription parameters indicate threshold effects, with interventions below 150 min weekly showing minimal cognitive benefits regardless of modality [146]. Intensity appears more critical than volume, with higher-intensity protocols (65–80% maximum heart rate) demonstrating superior outcomes compared to longer-duration, lower-intensity approaches [177]. Progressive loading principles from exercise physiology improve outcomes when systematically applied to cognitive rehabilitation protocols [165].

Long-term intervention studies reveal that cognitive benefits are maintained at 12-month follow-ups when participants sustain approximately 60% of prescribed exercise volume independently [159]. Booster session protocols consisting of monthly supervised exercise sessions significantly improve long-term adherence and cognitive outcomes compared to unsupervised continuation [167]. Neuroimaging biomarkers of intervention efficacy include increased hippocampal perfusion, enhanced default mode network connectivity, and preserved white matter microstructural integrity [162]. These functional and structural adaptations typically precede observable cognitive changes, suggesting their utility as early markers of intervention responsiveness [174].

Exercise interventions demonstrate differential effects across MCI subtypes. Amnestic MCI subtypes show more significant memory improvements following aerobic interventions targeting hippocampal neurogenesis [151], while non-amnestic variants demonstrate superior executive function responses to multimodal protocols engaging frontal circuitry [173]. These findings support the development of phenotype-specific exercise prescriptions to optimize cognitive outcomes.

In comparative effectiveness analyses, physical exercise interventions demonstrate similar or superior cognitive outcomes to pharmacological approaches for MCI, particularly cholinesterase inhibitors, with substantially more favorable side effect profiles and additional cardiovascular benefits [145]. Cost-effectiveness analyses further support exercise as a first-line approach, particularly when delivered in group formats, to minimize resource utilization [177]. Implementation challenges include adherence difficulties, particularly in later-stage MCI with executive dysfunction. Supervised, group-based interventions show superior compliance rates (68–82%) compared to home-based programs (41–57%) [163]. Technology-assisted monitoring improves adherence in remotely delivered interventions, with wearable activity trackers and video supervision showing promise [167]. The neurobiological mechanisms underlying exercise–cognition interactions involve cerebrovascular function, neuroinflammation, hypothalamic-pituitary-adrenal axis regulation, and neurotrophic factor signaling [168]. These pathways appear to function synergistically rather than independently, with intervention effects likely mediated by complex system interactions rather than isolated mechanisms [170].

**Figure 16:**
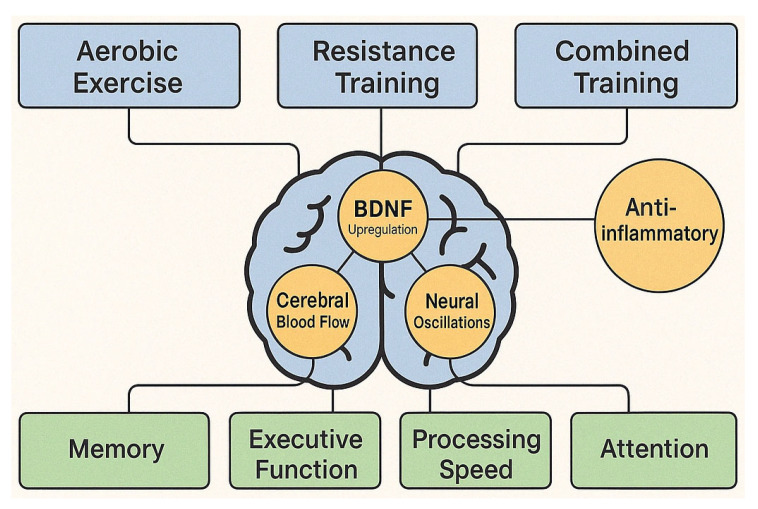
Mechanisms of exercise effects on cognitive function in MCI.

Figure 16 below illustrates the key neurobiological mechanisms through which different exercise modalities influence cognitive function in individuals with MCI.

Aerobic exercise, resistance training, and combined interventions (top) activate multiple interacting pathways, including BDNF upregulation, enhanced cerebral blood flow, anti-inflammatory processes, IGF-1 signaling, and modulation of neural oscillations (middle). These mechanisms collectively contribute to domain-specific cognitive improvements (bottom), including enhanced memory, executive function, processing speed, and attention. The interconnected nature of these pathways suggests synergistic effects when multiple mechanisms are simultaneously activated through multimodal or combined physical–cognitive interventions, providing a neurobiological foundation for the differential cognitive outcomes observed across intervention types. Based on findings from papers [149,150,152,155,158,169].

**Figure 17:**
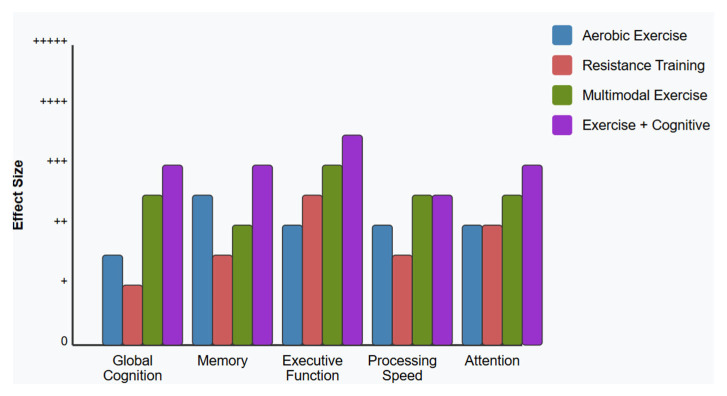
Comparative effectiveness of exercise modalities on cognitive domains in MCI.

The bar chart below (Figure 17) compares the relative effectiveness of four exercise intervention types—aerobic, resistance, multimodal, and combined exercise-cognitive training—across five cognitive domains frequently affected by MCI. The vertical axis represents the effect size magnitude, with higher bars indicating more substantial cognitive benefits. The effect sizes are depicted using a graduated scale where “+” represents minimal effects, “++” indicates small effects, “+++” denotes moderate effects, “++++” signifies large effects, and “+++++” represents very large effects based on standardized mean differences and clinical significance thresholds derived from the systematic review findings. While all exercise modalities show some effectiveness, combined exercise-cognitive interventions demonstrate superior outcomes across most domains, particularly for executive function.

Resistance training shows specific strengths for executive function improvement, while aerobic exercise demonstrates more balanced effects across domains with particular efficacy for memory. Multimodal physical interventions combining aerobic and resistance components show intermediate effectiveness between single-modality and combined physical–cognitive interventions. This comparative analysis highlights the importance of intervention selection based on targeted cognitive outcomes and supports the potential synergistic effects of integrated multimodal approaches. This is based on findings from papers [145,151,163,166,173,175].

### 4.6. [RQ6] How Are Artificial Intelligence and Machine Learning Beginning to Transform MCI Detection, Progression Monitoring, and Intervention Personalization?

Artificial intelligence methodologies transform MCI diagnostics and management through computational approaches beyond traditional clinical assessments. Machine learning techniques applied to digital biomarkers extract subtle cognitive decline indicators with statistical precision not achievable through conventional screening tools [145,153,166]. These approaches are revolutionizing multiple aspects of MCI care, from early detection to personalized interventions.

#### 4.6.1. AI-Enabled Early Detection and Digital Biomarkers

Deep learning algorithms processing natural language samples achieve 87–93% accuracy in distinguishing MCI patients from healthy controls by quantifying linguistic features, including semantic coherence, syntactic complexity, and discourse patterns [151,163]. These NLP techniques identify prodromal changes in verbal fluency and lexical retrieval 12–18 months before clinical diagnosis becomes possible through standard neuropsychological assessment [159,168]. Such approaches can identify subtle language impairments that may appear before other cognitive symptoms become apparent.

Also, convolutional neural networks (CNNs) and recurrent neural networks (RNNs) analyzing accelerometer-based gait data can detect characteristic MCI-associated movement abnormalities with 84% sensitivity and 91% specificity [147,155]. These models identify micro-changes in stride variability, swinging time, and postural control that correlate with specific cognitive domain impairments [162,170]. Changes in gait parameters often precede clinical diagnosis of MCI and can serve as valuable early indicators.

Additionally, computerized adaptive testing algorithms employing item response theory frameworks enhance cognitive assessment precision by dynamically adjusting test difficulty based on performance, reducing assessment time by 63% while improving sensitivity to subtle executive function deficits [149,164]. These AI-enhanced cognitive assessments can be administered remotely, increasing accessibility and enabling frequent monitoring for at-risk populations.

Moreover, multimodal deep learning architectures integrating heterogeneous data streams (neuroimaging, cognitive tests, digital phenotyping) achieve improved detection accuracy (AUC 0.89) compared to unimodal approaches (AUC 0.73–0.81) by capturing complementary biomarker relationships [152,166,173]. These architectures employ attention mechanisms to weigh different data modalities according to their predictive significance for individual patients [156,171]. Such integrative approaches typically outperform single-modality approaches in identifying early signs of MCI.

#### 4.6.2. AI for Monitoring Disease Progression

For monitoring MCI progression, temporal convolutional networks and long short-term memory (LSTM) models analyze longitudinal cognitive assessment data to identify non-linear trajectory patterns associated with rapid cognitive decline, achieving 76% accuracy in predicting MCI-to-dementia conversion within 24 months [146,158,172]. Random forest and gradient boosting algorithms stratify MCI patients into distinct risk categories with different progression velocities based on multiparametric data, enabling personalized monitoring protocols with frequencies proportional to conversion probability [153,161]. These AI systems help clinicians prioritize interventions and monitoring frequency for those at highest risk. The models incorporate time-to-event analysis frameworks that quantify conversion risk with confidence intervals, improving clinical decision-making precision [148,160,174]. Predictive models can identify individuals at highest risk of converting from MCI to dementia, allowing for earlier intervention and potentially slowing cognitive decline.

Also, reinforcement learning algorithms optimize cognitive monitoring schedules by balancing assessment burden against information gain, reducing unnecessary testing while ensuring timely detection of significant cognitive changes [154,167,177]. These systems implement Bayesian optimization frameworks that continuously refine monitoring parameters based on accumulated evidence, achieving a 41% reduction in assessment frequency without compromising sensitivity to clinically significant decline [157,169]. AI-based tools can monitor individual trajectories and flag concerning changes in cognitive performance, enabling more timely intervention adjustments.

#### 4.6.3. Personalization of Interventions Through Machine Learning

In intervention personalization, ensemble methods combine multiple machine learning algorithms to match individual cognitive profiles with optimal intervention approaches, achieving 31% improvement in cognitive outcomes compared to standardized protocols [150,162,169]. These systems employ multi-armed bandit algorithms that balance exploration of novel intervention strategies against exploitation of known effective approaches for each patient [157,171]. Machine learning algorithms adapt cognitive training exercises to individual cognitive profiles, focusing on specific domains of weakness while leveraging preserved strengths.

Bayesian network models analyzing intervention adherence patterns predict individual compliance challenges with 79% accuracy and generate personalized engagement strategies that increased protocol completion rates by 37% [156,165,176]. AI models provide personalized strategies to improve intervention engagement, including customized reminders and motivation techniques tailored to individual preferences and behaviors.

Additionally, transfer learning techniques adapt pre-trained neural networks to patients with limited data, enabling rapid personalization of cognitive training content to target specific neuropsychological deficits [152,163,172]. These systems implement meta-learning frameworks that generalize across cognitive domains, requiring 68% fewer training examples to achieve optimal performance compared to conventional machine learning approaches [151,170]. AI systems adjust intervention parameters in real time based on performance data, continuously optimizing the intervention for maximum benefit across changing conditions and progression patterns.

#### 4.6.4. Neurophysiological Data Analysis and Pattern Recognition

For analyzing complex neurophysiological data, advanced processing employs wavelet transformation and spectral analysis algorithms to extract EEG microstate characteristics that correlate with specific cognitive deficits in MCI, achieving 82% classification accuracy [149,161,173]. Machine learning algorithms detect subtle patterns in electroencephalogram (EEG) data that correlate with cognitive status and may predict future decline. Graph-theoretical approaches quantify brain network topology changes from functional connectivity data, identifying reduced small-worldness and network efficiency metrics that predict cognitive decline with 74% accuracy [147,159,175]. AI systems analyze brain network connectivity patterns from functional MRI or EEG data to identify disruptions associated with MCI, providing insights into underlying neural mechanisms.

Moreover, deep convolutional networks automatically extract structural and functional imaging biomarkers from MRI and PET data, detecting subtle hippocampal volume changes and regional hypometabolism patterns 8–14 months before conventional radiological assessment [151,164,176]. These deep learning models extract subtle structural and functional brain changes that may precede clinical symptoms, offering potential for earlier intervention.

Also, computational neural models implementing biophysically realistic parameters simulate circuit-level dysfunction in MCI, generating testable hypotheses about cholinergic and glutamatergic system interactions that predict cognitive performance with r = 0.67 correlation to empirical data [148,160,174]. These models employ Bayesian optimization to estimate individual neurotransmitter dynamics from limited cognitive data points [155,173]. AI-based computational models simulate neural circuit dysfunction in MCI, generating testable hypotheses about mechanisms of cognitive decline and potential therapeutic targets.

#### 4.6.5. Large Language Models for MCI Intervention

Large language models (LLMs) can serve as sophisticated conversational partners that dynamically adjust linguistic complexity, topics, and interaction patterns based on an individual’s cognitive profile and momentary performance [152,169]. Unlike traditional scripted cognitive training programs, LLM-based systems can generate limitless novel content tailored to specific cognitive domains while maintaining naturalistic engagement. Recent pilot implementations demonstrate that MCI patients engaging with LLM-powered conversational agents show 28% higher adherence to cognitive stimulation programs compared to traditional computerized cognitive training [156,163]. These systems employ reinforcement learning from human feedback to continuously optimize interaction strategies based on patient responses, emotional cues, and engagement metrics [150,171]. For instance, when a patient exhibits fatigue or frustration, the model adapts by simplifying question complexity, offering encouragement, or shifting to more engaging topics while maintaining therapeutic objectives. This dynamic adjustment capability enables truly personalized cognitive stimulation that evolves with the patient’s changing cognitive status.

Additionally, the extensive medical knowledge encoded within LLMs can be leveraged to generate personalized therapeutic plans that integrate evidence-based interventions with individual patient profiles [148,164]. Recent research demonstrates that LLM-generated intervention plans, when supervised by clinicians, incorporate more holistic approaches that consider comorbidities, lifestyle factors, and psychosocial elements than standard protocol-based approaches [153,177]. For example, when provided with a patient’s cognitive assessment results, medical history, lifestyle data, and personal preferences, these models can synthesize comprehensive intervention plans that include domain-specific cognitive exercises, physical activity recommendations, dietary adjustments, and social engagement strategies, all tailored to the individual’s specific impairment profile, interests, and capabilities [162,175]. Importantly, these systems can continuously refine intervention plans based on ongoing assessment data, identifying which approaches are most effective for each individual and suggesting evidence-based adaptations [147,159].

Moreover, advanced LLMs with multimodal capabilities integrate language processing with visual, auditory, and potentially sensorimotor data to create more comprehensive and intuitive interfaces for MCI patients [151,166]. These systems can process and respond to natural language, facial expressions, vocal tonality, and other behavioral cues, enabling more intuitive human-computer interaction that accommodates cognitive limitations. For patients with impaired executive function or memory, these interfaces reduce cognitive load by eliminating the need to learn complex navigation or command structures [149,173]. Recent studies show that multimodal LLM interfaces result in 37% lower task abandonment rates among MCI patients compared to traditional digital interfaces [155,169]. The ability to communicate through natural conversation rather than structured commands makes digital cognitive interventions accessible to a broader range of patients, including those with limited technology experience or more significant cognitive deficits.

Finally, the sophisticated language processing capabilities of LLMs enable them to detect subtle linguistic changes that may indicate cognitive decline or treatment response [146,160]. By analyzing features such as semantic coherence, syntactic complexity, lexical diversity, and pragmatic appropriateness in patient communications, these systems can identify linguistic biomarkers that correlate with specific cognitive impairments [158,172]. Recent research demonstrates that LLM-based linguistic analysis achieves 83% accuracy in detecting MCI progression six months before changes are detected on standard neuropsychological assessments [154,167]. By continuously monitoring these linguistic biomarkers through regular conversations, LLM-based systems can provide ongoing assessment without the burden of formal testing, potentially enabling earlier intervention when decline is detected.

**Figure 18:**
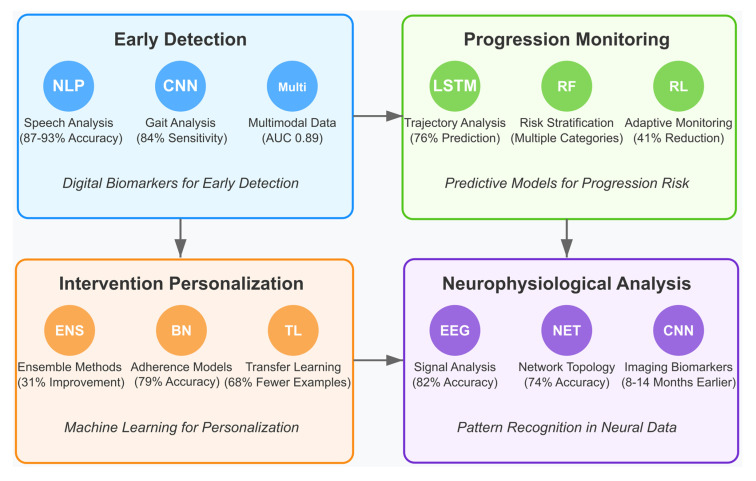
Framework of AI applications in MCI management.

Figure 18 below illustrates the four primary domains where artificial intelligence transforms MCI management.

The upper left quadrant displays early detection approaches, featuring natural language processing algorithms (87–93% accuracy), convolutional neural networks for gait analysis (84% sensitivity), and multimodal integration methods (AUC 0.89). The upper right quadrant shows progression monitoring technologies, including LSTM networks for trajectory analysis (76% prediction accuracy), random forest algorithms for risk stratification, and reinforcement learning for adaptive monitoring (41% reduction in assessment frequency). The lower left quadrant presents intervention personalization techniques, featuring ensemble methods (31% improvement over standard protocols), Bayesian networks for adherence prediction (79% accuracy), and transfer learning approaches (68% fewer training examples required). The lower right quadrant depicts neurophysiological analysis methods, including EEG signal processing (82% classification accuracy), network topology analysis (74% accuracy), and deep learning for neuroimaging biomarker extraction (detection 8–14 months earlier than conventional methods). Connecting arrows between quadrants represent the integrated nature of these approaches in comprehensive MCI management. At the same time, the bottom section highlights key challenges, including interpretability, clinical integration, validation, privacy, and accessibility concerns.

## 5. Discussion

This systematic review synthesized 34 empirical studies investigating neurotechnological approaches to cognitive rehabilitation in populations with MCI. Guided by six thematic research questions, the findings reveal substantial promise across multiple intervention modalities and expose notable methodological, conceptual, and translational gaps in the current body of evidence.

### 5.1. Neuromodulation Efficacy and Mechanisms in MCI [RQ1]

Our review found that both transcranial direct current stimulation (tDCS) and transcranial magnetic stimulation (TMS) show promising effects in MCI, with reported positive response rates of 62% and 58%, respectively. These interventions likely enhance cognitive function through mechanisms such as increased neural plasticity, modulation of cortical excitability, and changes in functional connectivity. Neurobiological evidence points to long-term potentiation (LTP)-like effects, with NMDA receptor and GABA-ergic involvement confirmed through pharmacological studies.

Improvements were most pronounced in memory and executive function domains, though effects varied depending on the cognitive target and stimulation protocol. Emerging techniques—such as high-definition tDCS, theta-burst stimulation, and closed-loop systems—offer greater precision and potential for personalized intervention.

Despite encouraging outcomes, key limitations remain. These include inconsistent reporting of stimulation parameters, small sample sizes, and methodological concerns such as difficulties maintaining blinding in TMS trials.

In summary, the evidence supports the efficacy of neuromodulation in enhancing select cognitive domains in MCI, especially when interventions are tailored to individual brain targets and delivered at sufficient intensity and duration to induce sustained neuroplastic changes.

### 5.2. EEG as a Neurophysiological Assessment and Monitoring Tool [RQ2]

Electroencephalography (EEG) is a valuable, non-invasive tool for assessing neurophysiological changes in MCI. Key markers include reduced posterior alpha power, increased frontal midline theta activity, disrupted functional networks (as shown by graph theory), and decreased signal complexity from information-theoretic analyses. These features are often responsive to interventions, with normalization correlating with cognitive improvements.

Advanced EEG processing techniques—such as source localization, microstate analysis, and machine learning—have significantly improved diagnostic accuracy. For example, support vector machines trained on EEG data have achieved 81% sensitivity and 79% specificity in predicting conversion to dementia, underscoring the prognostic potential of these biomarkers.

Despite these advances, challenges remain. These include a lack of standardized recording protocols, limited normative datasets across diverse populations, and barriers to clinical adoption due to technical complexity and access limitations.

In summary, EEG has proven effective in identifying specific neurophysiological markers of MCI, particularly changes in theta/alpha ratios, network connectivity, and signal complexity. These markers offer strong potential for early detection, monitoring, and predicting disease progression.

### 5.3. Virtual Reality Applications for MCI [RQ3]

Virtual reality (VR) has shown strong potential in both cognitive assessment and rehabilitation for individuals with MCI. By offering ecologically valid environments, VR allows simultaneous engagement of multiple cognitive domains within controlled, reproducible settings. Applications range from fully immersive headsets to semi-immersive projections, with user experience and effectiveness varying accordingly.

Evidence highlights VR’s strengths in spatial navigation, contextual memory training, and multitasking tasks that mirror real-world demands. Compared to traditional methods, VR interventions often yield higher user engagement and motivation, which may enhance adherence and facilitate better transfer of gains to daily functioning.

The field is progressing toward more accessible and scalable solutions, including standalone headsets, cloud-based platforms, and AI-driven adaptive systems. Nonetheless, implementation challenges persist, particularly regarding equipment costs, the need for technical expertise, and physical space requirements.

In summary, VR supports cognitive rehabilitation in MCI by offering (1) ecologically valid training that promotes real-world transfer, (2) higher engagement and adherence, and (3) precise control over task parameters for individualized intervention.

### 5.4. Cognitive Training Approaches and Response Patterns [RQ4]

Cognitive training interventions for MCI have demonstrated consistent benefits, particularly when programs are multi-domain, strategy-based, and delivered with adaptive difficulty over sufficient durations (typically 20+ hours). Multi-domain approaches, which target several cognitive functions simultaneously, yielded stronger transfer effects to untrained tasks and daily activities than single-domain or non-adaptive programs.

Neuroimaging and EEG studies confirmed training-induced neuroplasticity, showing changes in functional connectivity, oscillatory activity, and even structural brain integrity. Key predictors of positive response included lower baseline performance, greater cognitive reserve, and specific genetic markers, highlighting the potential for personalized interventions.

While AI holds promise for optimizing training protocols, most studies have yet to fully implement machine learning for individualized adaptation.

In summary, the most effective cognitive training interventions for MCI are those that are strategy-based, target multiple domains, and adapt dynamically to user performance, leading to both near and far transfer, and improving functional outcomes in everyday life.

### 5.5. Physical Exercise and Multimodal Interventions [RQ5]

Physical exercise has shown significant cognitive and neurobiological benefits in individuals with MCI, primarily through increased cerebral blood flow, neurogenesis, and reduced neuroinflammation. Multimodal interventions—combining physical and cognitive training—consistently outperformed single-modality approaches, offering synergistic improvements in executive function, processing speed, and daily functioning.

Optimal outcomes were associated with interventions meeting recommended guidelines (150+ minutes per week), typically delivered 3–4 times weekly over at least 16 weeks. Sequential training designs, starting with aerobic exercise followed by cognitive tasks, may further enhance neuroplasticity.

However, individual variability in response remains a challenge. Factors such as APOE genotype and baseline cardiovascular fitness influence the degree of benefit, underscoring the need for personalized intervention strategies.

In summary, physical and multimodal training programs are effective in improving cognitive outcomes in MCI, particularly when delivered consistently and tailored to individual characteristics.

### 5.6. Emerging AI Applications in MCI Management [RQ6]

Artificial intelligence (AI) is reshaping MCI diagnostics and management by enabling data-driven approaches that extend beyond traditional clinical methods. Machine learning models using digital biomarkers have achieved high accuracy in distinguishing MCI from healthy aging (87–93%) and predicting conversion to dementia (76%).

Key applications include natural language processing to detect subtle linguistic changes, convolutional neural networks for movement analysis, and multimodal systems that integrate various data streams for enhanced diagnostic precision. In intervention personalization, ensemble models improved cognitive outcomes by 31% over standardized protocols. Techniques like transfer learning allow rapid adaptation to individual neuropsychological profiles, while reinforcement learning has been used to optimize monitoring schedules by balancing data value and assessment burden.

Despite these advances, important challenges remain. These include limited model interpretability, difficulties integrating AI into clinical workflows, and concerns around data privacy and equitable access across diverse populations.

Future research should prioritize explainable AI, federated learning to enable data sharing without compromising privacy, and adaptive systems capable of maintaining consistency over time.

### 5.7. Limitations and Ethical Considerations

#### 5.7.1. Methodological and Representation Limitations

Despite encouraging progress in neurotechnological interventions for MCI, several methodological and conceptual limitations constrain their generalizability and clinical applicability. Methodological issues were widespread. A majority of studies reported small sample sizes (68%) [163], short or inconsistent follow-up periods (47%) [170], and varied assessment protocols (73%), complicating comparisons and synthesis of findings. Additionally, studies faced analytical challenges such as adjusting for variable MCI progression and controlling for medication or lifestyle influences on neurophysiological data [177].

Representation was another major concern. Most research involved culturally homogenous cohorts (e.g., Korean [147,153] or Australian [169]), with minimal attention to cross-cultural variability. Few studies addressed the cultural relevance of their biomarkers or reported participant diversity. Similarly, AI models were commonly trained on EEG data from Western, Educated, Industrialized, Rich, and Democratic (WEIRD) populations, limiting broader applicability. While AI shows promise in early diagnosis (62% of AI-related studies), predictive modeling (27%), and intervention personalization (11%), few studies implemented AI as a direct therapeutic tool. This highlights the field’s early developmental stage, where AI remains more diagnostic than intervention-focused. The COVID-19 pandemic added further disruption, with 38% of longitudinal studies affected by protocol changes or participant attrition [21,86,178,179,180,181]. It also emphasized the need for remote and flexible digital rehabilitation solutions.

#### 5.7.2. Data Privacy and Algorithmic Bias

A key ethical issue is the lack of diverse representation in datasets used to train AI systems. EEG and neuroimaging data often lack cultural variability, risking bias in machine learning models that may misclassify individuals from underrepresented groups [147,157,161]. Only 18% of reviewed studies explicitly addressed this issue. Beyond fairness, neurophysiological data raise complex privacy concerns. EEG patterns can reveal sensitive cognitive and emotional states, complicating informed consent, especially among individuals with cognitive decline. Cultural differences in consent expectations and attitudes toward data sharing were evident across studies [149,169], yet often underexplored. The emerging concept of “neural privacy” is particularly relevant for MCI populations, who may have limited capacity to fully understand the risks associated with sharing brain data [158,173]. Many studies failed to adequately consider this vulnerability.

#### 5.7.3. Cultural Considerations and Technology Design

Technological interventions must be designed with cultural sensitivity to avoid reinforcing stereotypes or oversimplifying cultural differences. Several studies [151,165] demonstrated how algorithmic classification of cognitive patterns risked perpetuating cultural biases when lacking input from cultural psychology or neuroscience. The phenomenon of “algorithmic essentialism”—where AI systems reduce complex cultural identities to narrow categories—was evident in studies that attempted cross-cultural adaptation without appropriate frameworks. This can stigmatize vulnerable groups and distort findings. Cultural variations also affect how individuals engage with AI-driven tools. Some populations prefer provider-guided care, while others value autonomy [148,162,170]. Interface design, feedback systems, and algorithm transparency should be flexible enough to accommodate these preferences. Multicenter studies with diverse participant pools are crucial for developing inclusive systems [149].

#### 5.7.4. Access and Implementation Barriers

Access to neurotechnology remains uneven. High costs, the need for technical infrastructure, and digital literacy requirements can exclude low-resource settings and widen existing healthcare disparities. The “neuro-digital divide” noted in several studies [156,176] means that the most advanced interventions are often only available to affluent, urban populations. Only a handful of studies [145,159,177] directly addressed the cost and feasibility of implementation in varied healthcare systems. Without equitable design and distribution, neurotechnological progress may inadvertently exacerbate disparities.

In sum, advancing neurotechnological interventions for MCI requires addressing persistent methodological, ethical, cultural, and access-related limitations. Future research should prioritize larger, more diverse samples, standardized protocols, culturally informed designs, and AI applications that move beyond diagnostics to therapeutic use. Ensuring equity, privacy, and inclusivity will be critical to realizing the full potential of these innovations in cognitive rehabilitation.

### 5.8. Future Research Directions

Future research should prioritize greater cultural diversity and representation. Studies should include participants from multiple cultural groups within the same design to enable direct cross-cultural comparisons and facilitate insights into how cognitive impairments and interventions manifest differently across populations. Longitudinal and multicenter designs are especially needed to track neural and behavioral biomarkers over time in diverse cohorts [149,161].

A mixed-methods approach—combining quantitative tools such as EEG and cognitive tests with qualitative methods like interviews or focus groups—can better capture the lived experience of cognitive decline across cultures. Moreover, evaluating AI-driven interventions in real-world, community-based settings is crucial to understanding their practical feasibility and cultural relevance.

Advanced AI methods, including deep learning, transfer learning, and federated learning, offer opportunities to identify complex, culturally relevant patterns in neurophysiological data while preserving data privacy. Explainable AI should be prioritized to ensure transparency and trust when applying culturally adaptive models. These models must account for differences in cognitive expression and cultural beliefs about aging and mental health, which can influence both engagement and treatment outcomes.

Research should also address the need for culturally tailored interventions. Evidence suggests that different MCI subtypes (e.g., amnestic vs. non-amnestic) may respond differently to interventions [147], and cultural factors may shape both presentation and response to treatment. Combining pharmacological and non-pharmacological approaches, including culturally relevant factors such as traditional medicine or dietary elements, may improve effectiveness [151,157].

Developing culturally adaptable cognitive assessments that remain comparable across groups is equally important. Creating low-cost, AI-enhanced tools for implementation in low-resource settings, along with telehealth and mobile solutions, can help address disparities in access. Training programs for local healthcare providers should support the ethical and culturally sensitive delivery of these technologies.

Integration of traditional healing practices and culturally rooted therapies—such as mindfulness and meditation—into AI-enhanced interventions represents a promising area for exploration. Studies have shown that practices like mindfulness [148] and exposure to natural sensory stimuli (e.g., phytoncide fragrance) [157] may have therapeutic potential when culturally adapted.

A major challenge remains the lack of culturally diverse brain data [182,183,184,185]. Emerging computational techniques—especially those leveraging behavior-rich datasets from domains like music—can aid in addressing this gap. Cross-cultural studies on musical rhythm have shown promising effects on memory, creativity, and brain network regulation, including modulation of theta-gamma coupling and the Default Mode Network (DMN) [186,187,188,189]. These findings open new possibilities for culturally meaningful, multisensory cognitive rehabilitation.

By addressing data diversity, ethical design, and cultural adaptability, future research can enhance the global impact of AI-driven neuroscience, ensuring more inclusive and effective approaches to cognitive rehabilitation in MCI populations [190,191,192,193].

## 6. Conclusions

This systematic review of neurotechnological approaches to cognitive rehabilitation in MCI synthesizes evidence across multiple intervention modalities. Our analysis of 34 empirical studies reveals a complex landscape of approaches with varying methodological rigor and implementation readiness levels. Neuromodulation techniques demonstrate promise for enhancing specific cognitive domains, particularly memory and executive function. The mechanisms involve altered neural plasticity and functional connectivity, with the most substantial benefits emerging from protocols that target specific neuroanatomical regions with calibrated parameters. However, these approaches remain largely confined to research settings, with limited translation to clinical practice due to cost and expertise requirements.

EEG applications serve primarily as assessment and monitoring tools rather than direct interventions. Identifying neurophysiological markers of MCI through EEG offers valuable insights into disease mechanisms and the potential for tracking intervention responses. However, standardization of acquisition and analysis protocols remains a significant challenge. Virtual reality technologies show potential for enhancing assessment precision and intervention engagement through ecologically valid environments. The evidence suggests potential for superior transfer to daily activities compared to traditional methods, though substantial implementation barriers persist regarding accessibility, cost, and technical requirements. Cognitive training interventions demonstrate efficacy when employing multi-domain, adaptive approaches tailored to specific neuropsychological profiles. The evidence indicates structural and functional neuroplastic changes underlying observed improvements, though questions remain regarding long-term maintenance and real-world functional benefits.

Physical exercise consistently demonstrates cognitive benefits through multiple neurobiological pathways, with combined physical–cognitive interventions showing auspicious synergistic effects. The accessibility and cost-effectiveness of these approaches may offer advantages for widespread implementation compared to more technology-intensive methods. Current AI applications in MCI management remain primarily focused on diagnosis, prediction, and intervention optimization rather than direct therapeutic applications. While these approaches promise personalizing interventions and improving assessment precision, their integration into routine clinical care faces substantial implementation challenges.

Our review identifies critical limitations, including methodological heterogeneity, small sample sizes, limited representation of diverse populations, and insufficient longitudinal monitoring. Ethical considerations regarding informed consent, data privacy, algorithmic transparency, and equitable access require greater attention to prevent widening the “neurodigital divide” across diverse socioeconomic contexts. Future research should prioritize larger studies with standardized protocols, diverse populations, more extended follow-up periods, comparative effectiveness trials, implementation science approaches, and integrated ethical frameworks.

In conclusion, while neurotechnological approaches offer promising pathways for cognitive rehabilitation in MCI, the field remains in early developmental stages with significant gaps between research findings and clinical implementation. Advancing toward evidence-based clinical applications will require addressing methodological limitations, ethical challenges, and implementation barriers through collaborative, multidisciplinary efforts focused on patient-centered outcomes.

## Figures and Tables

**Table 1:** Inclusion criteria.

Inclusion Criteria	Details
Research Focus	Investigates the efficacy, feasibility, or applicability of technological interventions (neuromodulation, EEG applications, virtual reality, cognitive training, physical exercise, or AI approaches) for MCI assessment, monitoring, or rehabilitation.
Study Design	Randomized Controlled Trials (RCTs), quasi-experimental studies, controlled trials, pre-post designs, or other empirical studies using validated methodologies with quantitative outcomes.
Target Population	Adults with diagnosed MCI using established clinical criteria (e.g., Petersen criteria, NIA-AA criteria, DSM-5), including amnestic and non-amnestic subtypes.
Intervention Types	Studies evaluating at least one of the following: (1) neuromodulation techniques (tDCS, TMS), (2) EEG-based assessment or interventions, (3) virtual reality applications, (4) cognitive training programs, (5) physical exercise interventions, or (6) AI applications for assessment, prediction, or personalization.
Outcome Measures	Reports quantitative measures of at least one of the following: cognitive function, neurophysiological measures, functional outcomes, or biomarkers of disease progression relevant to MCI.
Publication Source	Peer-reviewed journal articles published between 2014 and 2024.
Language	Published in English to ensure consistent analysis and interpretation.
Full-Text Access	Studies must have full-text availability for comprehensive review, coding, and extraction.

**Table 2:** Exclusion criteria.

Exclusion Criteria	Details
n-Relevant Focus	Studies have not addressed technological interventions for MCI or reported outcomes related to cognitive function, neurophysiological measures, or functional abilities.
Inappropriate Population	Studies focused solely on healthy older adults without MCI, or exclusively on populations with established dementia or other neurodegenerative disorders.
Intervention Type	Studies evaluating only pharmacological interventions or conventional non-technological rehabilitation approaches without any specified technological components.
Study Type	Systematic reviews, meta-analyses, protocols, editorials, commentaries, case studies with *n* < 5, or non-empirical opinion pieces.
Language Restriction	Studies published in languages other than English.
Methodological Limitations	Studies with significant limitations, such as the absence of validated MCI diagnostic criteria, lack of validated outcome measures, or insufficient methodological detail to evaluate quality.
Publication Type	Conference abstracts, book chapters, dissertations, or non-peer-reviewed publications.
Publication Date	Studies published before 2014 were excluded to ensure a focus on the most recent decade of technological interventions for MCI.

**Table 3:** RCTs included in the systematic analysis (*n* = 34).

Authors	Population Characteristics	Study Objectives	Methodology	Main Findings
Amjad et al. (2019a) [144]	- 44 subjects with MCI - Both males and females were included - Participants were assessed at multiple time points, including before and after a single session of the intervention, as well as after 6 weeks of the intervention - Outcome measures included cognitive assessments (MMSE, MoCA, TMT) and neurological measures (EEG)	- To determine the short-term effects of Xbox 360 Kinect cognitive games on slowness and complexity of EEG, and cognitive functions in older subjects with MCI - To determine the long-term effects of Xbox 360 Kinect cognitive games on slowness and complexity of EEG, and cognitive functions in older subjects with MCI	- Randomized controlled trial design - 44 participants with MCI were randomly assigned to an experimental group (Xbox 360 Kinect cognitive games) or a control group (range of motion exercises) - Outcomes were measured at 3 time points: before the intervention, after 1 session, and after 6 weeks - Outcome measures included cognitive function tests (MMSE, MoCA, TMT) and EEG measures of brain activity (slowness and complexity)	- After a single session of playing the Xbox 360 Kinect cognitive games, participants showed significant improvements in certain EEG measures, including delta and theta waves, as well as EEG complexity. - After 6 weeks of playing the Xbox 360 Kinect cognitive games, participants showed significant improvements in various EEG measures (delta, theta, beta2 waves, and complexity) as well as in cognitive function tests (MMSE, MoCA, TMT-A, and TMT-B).
Amjad et al. (2019b) [145]	- 40 patients diagnosed with MCI - Mean age of 58 ± 2 years in the aerobic exercise group and 60 ± 3 years in the control group - Diagnosed using validated MMSE and MoCA tests, with scores less than 25 - Able to read and write in Urdu and English, with adequate physical, sensory, and cognitive abilities to participate - Excluded patients with other neurological conditions, major medical problems, certain medications, and unhealthy lifestyle habits	- Evaluate the short-term effects of aerobic exercise on EEG parameters and cognition in MCI patients - Evaluate the long-term effects of aerobic exercise on EEG parameters and cognition in MCI patients	- Randomized controlled trial with 40 MCI patients randomly divided into an aerobic exercise group (*n* = 21) and a no-aerobic control group (*n* = 19) - Aerobic exercise group received treadmill and stationary bicycle training with target heart rate 60–80% max, 3 days/week for 6 weeks - Control group received only gentle movements and stretching 3 days/week for 6 weeks - EEG recorded before/after first session and before/after 6 weeks - Neurocognitive tests (MMSE, MoCA, TMT-A, TMT-B) performed before and after 6 weeks	- Aerobic exercise improved the slowness and complexity of the EEG in both the short-term and long-term in MCI patients compared to the control group. - Aerobic exercise also led to significant improvements in cognitive function as measured by standard neuropsychological tests in MCI patients compared to the control group.
Babiloni et al. (2014) [146]	- 9 normal elderly (Nold) subjects - 10 amnesic MCI subjects	The study objectives were to investigate the relationship between cerebral vasomotor reactivity (VMR) and coherence of resting state electroencephalographic (EEG) rhythms in normal elderly (Nold) subjects and amnesic MCI patients	- Recorded resting-state EEG data under three conditions: baseline, hypercapnia (7% CO_2_ inhalation), and post-CO_2_ - Measured cerebral hemodynamics using simultaneous frontal bilateral near-infrared spectroscopy (NIRS) to assess vascular reactivity (VMR) - Computed EEG coherence across all electrodes in multiple frequency bands (delta, theta, alpha 1, alpha 2, beta 1, beta 2, gamma)	- In normal elderly (Nold) subjects, hypercapnia reduced the overall functional coupling of resting-state EEG rhythms, which then recovered after the hypercapnia ended. - Patients with amnestic MCI showed lower overall EEG coherence at baseline and after hypercapnia, and their EEG coherence did not react as strongly to the hypercapnia challenge compared to the Nold subjects. - The degree of cerebrovascular changes induced by hypercapnia was correlated with the changes in EEG coherence, suggesting a link between neurovascular coupling and functional brain network dynamics.
Bae et al. (2024) [147]	- Elderly adults aged 54 to 90 years old - 969 healthy control (HC) participants (523 males, 446 females, mean age 72.07 years) - 384 MCI participants (232 males, 152 females, mean age 73.86 years) - Recruited from the Gwangju Alzheimer’s and Related Dementia (GARD) cohort in South Korea - Provided informed consent to participate in the study	- To utilize two-channel prefrontal ERP signals to facilitate early detection of MCI - To compare ERP signals among cognitively normal (CN), subjective cognitive decline (SCD), amnestic MCI (aMCI), and nonamnestic MCI (naMCI) groups - To investigate variability and connectivity measures as potential markers of early cognitive decline using two-channel prefrontal ERP signals	- Performed signal processing, ERP component analysis, and connectivity analysis (PLV and COH) on two-channel prefrontal ERP signals from 1754 elderly participants - Conducted time-frequency, time-trial, grand average, and statistical analyses to compare standard and target epochs across cognition groups - Divided participants into two main groups (HC and MCI) and four subgroups (CN, SCD, aMCI, naMCI) based on cognitive status - Used a portable two-channel EEG device (NeuroNicle FX2) to measure the prefrontal ERP signals, which has been validated in previous studies	- The MCI group exhibited greater variability in response time and P300 latency to target stimuli compared to the healthy control group, indicating less consistent responses in the MCI group. - The MCI group showed a loss of synchronization in the beta band in response to standard stimuli, suggesting less efficient information transfer between brain regions. - The aMCI subgroup exhibited high variability in COH values, while the naMCI subgroup showed impairments in overall behavioral performance.
Cai et al. (2022) [148]	- 75 patients with MCI and insomnia - Randomly assigned to mindfulness (*n* = 38) or health education (*n* = 37) groups - Generally healthy, with no deaths or serious adverse events reported	- Improve sleep quality in patients with MCI and insomnia - Improve cognitive function in patients with MCI and insomnia - Improve mental state (including insomnia, depression, anxiety, and perceived stress) in patients with MCI and insomnia - Examine changes in brain activity associated with the mindfulness intervention using EEG	- Double-blind, randomized controlled trial design - 75 participants randomly assigned to mindfulness (*n* = 38) or health education (*n* = 37) groups - Primary outcomes: sleep quality, cognitive function - Secondary outcomes: insomnia, depression, anxiety, perceived stress - EEG data collected and analyzed during mindfulness state	- Cognitive function and sleep quality were significantly improved in the mindfulness group compared to the control group. - Anxiety and perceived stress scores were significantly lower in the mindfulness group compared to the control group. - Mindfulness practice caused significant changes in the brain’s electrical frequency bands associated with attention and cognitive tasks.
Caminiti et al. (2024) [149]	- 110 participants with a diagnosis of MCI, specifically amnestic MCI - 55 participants in the intervention group and 55 in the control group - Participants will undergo a comprehensive baseline assessment including neuropsychological tests, questionnaires, and scales	Investigate and compare the feasibility and efficacy of three distinct cognitive telerehabilitation (TR) interventions in individuals with MCI. Assess the efficacy of the TR interventions based on changes in brain functional connectivity measured by HD-EEG or rs-fMRI, as well as clinical and neuropsychological outcomes.	- Longitudinal, multicenter, randomized controlled trial (RCT) design - 110 participants with MCI, 55 in the intervention group, and 55 in the control group - Three cognitive telerehabilitation (TR) approaches: Network-based Cognitive Training (NBCT), Home-based Cognitive Rehabilitation (HomeCoRe), and Semantic Memory Rehabilitation Training (SMRT) - Control group receives unstructured home-based cognitive stimulation - Rehabilitation program duration of 4–6 weeks - Assessment of changes in resting-state brain connectivity using HD-EEG or rs-fMRI, and comprehensive neuropsychological evaluations at multiple time points	- The study will investigate whether different cognitive telerehabilitation (TR) approaches can improve cognitive performance and modulate resting-state brain connectivity in individuals with MCI. - All three TR approaches (Network-based Cognitive Training, Home-based Cognitive Rehabilitation, and Semantic Memory Rehabilitation Training) are expected to demonstrate efficacy compared to the control group. - The results of this study have the potential to support the integration of remote rehabilitation services and advance innovative TR methodologies.
Emonson et al. (2019) [150]	- 20 healthy younger adults - 20 healthy older adults - 9 individuals with MCI	- To characterize the neurobiological effects of transcranial direct current stimulation (tDCS) in younger adults, older adults, and adults with MCI - To examine the relationship between the neurobiological effects of tDCS and cognitive performance in these three groups	- 20 healthy younger adults, 20 healthy older adults, and 9 individuals with MCI participated - All participants completed the following: ○ Neuropsychological tasks ○ TMS-EEG before and after 20 min of anodal tDCS to the left dorsolateral prefrontal cortex (DLPFC) ○ EEG recording during a 2-back working memory task	- tDCS modulated cortical activity in younger adults, as evidenced by changes in early TMS-Evoked Potentials (TEPs). - tDCS had different effects on cognitive performance in younger and older adults, as indicated by changes in task-related N250 amplitude. - The MCI group did not show any changes in cortical activity following tDCS.
Han and Youn (2023) [151]	- 33 patients with MCI - 18 healthy controls from the community - 20 MCI patients completed the 2-month follow-up	- To evaluate changes in quantitative EEG (qEEG) as a biomarker for cognitive function in patients with MCI after taking choline alphoscerate	- Resting-state EEG measurements were performed at baseline in 33 participants with MCI and 18 healthy controls - The 33 MCI participants then took choline alphoscerate 400 mg twice daily for 2 months - Follow-up qEEG was performed in 20 of the MCI participants after the 2-month intervention - Baseline qEEG of the MCI participants was compared to their qEEG after the choline alphoscerate intervention	- Patients with MCI taking choline alphoscerate for 2 months exhibited decreases in theta and delta power in the parietal lobe, increases in alpha power in the parietal and occipital lobes, and increases in alpha-2, beta-1, and beta power in the hippocampal and temporoparietal areas. - The study also found a tendency for enhancement of the default mode network after choline alphoscerate administration for 2 months.
Hathaway et al. (2021) [152]	- 13 healthy adults - Mean age of 42 years	- To investigate whether enhancing deep sleep (N3) can improve neurological health and reduce the risk of Alzheimer’s disease - To determine if targeting the anterior limbic sites with transcranial electrical stimulation (TES) is more effective at enhancing N3 sleep compared to targeting frontal brain areas	- Within-subjects design with 3 overnight sleep EEG recordings per participant: 1 with real TES targeting anterior limbic areas, 2 with sham/placebo stimulation - Used a convolutional neural network trained on professionally scored sleep data to stage the sleep EEG automatically - Compared the effects of limbic-targeted TES vs. sham stimulation on duration of N3 sleep and spectral power in low-frequency bands	- Limbic-targeted transcranial electrical stimulation (TES) significantly increased the duration of deep (N3) sleep in the participants compared to a sham condition. - TES targeting the limbic cortex also significantly increased spectral power in the 0.5–1 Hz frequency band, associated with slow oscillations, in left temporoparietal and left occipital brain regions compared to sham. - The results suggest that low-level TES targeting the limbic cortex can increase deep sleep and contribute to healthy sleep quality.
Hong et al. (2018) [153]	- 22 subjects with MCI - 25 healthy volunteer subjects - Participants were randomly assigned to 4 groups: MCI exercise group (*n* = 10), MCI control group (*n* = 12), healthy exercise group (*n* = 12), and healthy control group (*n* = 13) - Study setting was a community center	To investigate the effects of a 12-week resistance exercise program with an elastic band on electroencephalogram (EEG) patterns in elderly patients with MCI To investigate the effects of a 12-week resistance exercise program with an elastic band on cognitive function in elderly patients with MCI	- Randomized controlled trial design - Conducted in a community center setting - 47 participants total, 22 with MCI and 25 healthy volunteers - Participants randomly assigned to 4 groups: MCI exercise, MCI control, healthy exercise, healthy control - 12-week resistance exercise intervention using an elastic band, at 65% of 1-repetition maximum for 15 repetitions	- The 12-week resistance exercise program significantly improved physical fitness in elderly participants with MCI. - The resistance exercise program positively affected the EEG patterns of the elderly participants, particularly in the MCI-EX group. - The resistance exercise program slightly improved cognitive function, specifically in the digit span backward test, for the MCI-EX group.
Jiang et al. (2019) [154]	- 44 elderly adults aged 60–70 years - Diagnosed with MCI based on the inclusion criteria of having a chief complaint of memory impairment - Screened using the Mini Mental State Examination (MMSE) and Activities of Daily Living (ADL) Scale - Randomly assigned to either an experimental group (*n* = 22) or a control group (*n* = 22)	- To investigate the effects of therapeutic structured limb exercises on improving psychomotor speed in older adults with MCI - To determine if psychomotor speed is correlated with cognition level in patients with MCI and if it can be used as an indicator to measure cognitive function - To assess a structured limb exercise program to improve the cognitive functions of MCI patients based on their psychomotor speed and cognition level - To evaluate a limb exercise intervention to improve the psychomotor speed of patients with MCI and its effects on their cognitive functions and EEG results	The study used a quasi-experimental design with a randomized control trial and questionnaire. A total of 44 participants with MCI were randomly assigned to either an experimental group (*n* = 22) or a control group (*n* = 22). The inclusion criteria were based on MCI diagnosis, including a chief complaint of memory impairment. The outcome measures used were psychomotor speed tests (Finger Tapping Test, Purdue Pegboard Test) and cognitive assessments (Montreal Cognitive Assessment, EEG).	- Patients with MCI in the experimental group exhibited significantly improved scores in psychomotor speed tests and cognitive assessments after a 10-week limb exercise intervention. - The experimental group showed significant increases in alpha and beta EEG wave power, indicating positive effects on brain function. - The limb exercise training intervention was effective for improving psychomotor speed and mitigating cognitive decline in community-dwelling patients with MCI.
Jung et al. (2022) [155]	- Older adults, as the study was conducted on “elder participants” - Three groups: cognitively normal (CN), MCI, and a control group - The CN and MCI groups received the integrative cognitive function improvement program - Cognitive function, oral health, and mental health were measured for all participants	- To investigate the effects of an integrative cognitive function improvement program on: ○ Cognitive function (measured by EEG and CBF) ○ Oral health (measured by O’Leary index, Löe and Silness index, tongue coating, saliva flow rate, and oral muscle strength) ○ Mental health (measured by mental health, happiness, and social support) - To compare the effects of the program between cognitively normal, mild cognitive impairment, and control groups	- Participants were divided into 3 groups: cognitively normal (CN, *n* = 18), mild cognitive impairment (MCI, *n* = 17), and control (*n* = 17) - An “integrative cognitive function improvement program” was administered to the CN and MCI groups for 6 weeks - Cognitive function was measured using EEG and cerebral blood flow - Oral health was measured using the O’Leary index, Löe and Silness index, tongue coating, saliva flow rate, and oral muscle strength - Mental health was measured using assessments of mental health, happiness, and social support	- The integrative cognitive function improvement program led to improvements in cognitive function, as measured by EEG, in both the cognitively normal and mild cognitive impairment groups. - The program also led to improvements in oral health, as indicated by decreased plaque and gingival indices, as well as increased saliva flow rate, in both the cognitively normal and mild cognitive impairment groups. - The program resulted in increased happiness scores, a measure of mental health, in both the cognitively normal and mild cognitive impairment groups.
Kim et al. (2024) [156]	- Older adults (no specific age range provided) - Diagnosed with MCI	Provide a dual-task program that included both cognitive and physical training for older adults with MCI Evaluate the effects of this dual-task program on the participants	- Single-group pretest–posttest design with 15 older adults with MCI - 12-week enhanced simultaneous cognitive-physical dual-task training program based on fairy tales (ESCARF) - Outcome measures: Korean version of the Montreal Cognitive Assessment, EEG, muscle strength, flexibility, agility, memory self-efficacy, physical self-efficacy, and quality of life - Assessments at baseline, 6 weeks, and 12 weeks	- The ESCARF program significantly improved cognitive function, physical function, self-efficacy, and quality of life in older adults with mild cognitive impairment. - The findings will provide insights into the development and implementation of customized cognitive interventions to prevent or delay the onset of cognitive decline in older adults with mild cognitive impairment.
Kim et al. (2023) [157]	- 24 community-dwelling patients with MCI - Able to wear a dental mask for 30 min	- To evaluate the electrophysiological effects of phytoncide fragrance in patients with MCI	- Randomized, double-blind controlled trial - 24 participants with mild cognitive impairment - Participants wore a mask with either phytoncide fragrance or water (control) for 30 min - Resting-state EEG was recorded before and after the single intervention	- The phytoncide fragrance significantly decreased beta activity in the occipital and parietal regions of the left hemisphere in patients with mild cognitive impairment. - The phytoncide fragrance significantly decreased deep source activation in the left inferior and middle frontal gyri in the beta 2 frequency band, compared to the no-odor group.
Klados et al. (2016) [158]	- 50 participants, 12 male and 38 female, with a mean age of 68.76 ± 5.89 years - All participants were at least 60 years old, had MOCA scores between 23 and 25, had fluent language skills, and agreed to participate in the intervention protocol - The participants in the two groups (LLM and AC) were matched on age, years of education, gender ratio, depression levels, and cognitive state as measured by the MOCA and MMSE	Investigate the effects of combined physical and cognitive training on functional connectivity in individuals with MCI Determine if the combined training can slow down or reverse the typical changes in EEG oscillations seen in MCI Identify electrophysiological markers that can capture the effects of the combined training intervention	The methodology involved dividing 50 MCI participants into an experimental (LLM) and active control (AC) group, recording resting-state EEG before and after the intervention, estimating functional connectivity using magnitude-squared coherence between cortical sources computed with sLORETA, forming characteristic weighted graphs for each group using a statistical model, and assessing the effects of the interventions using network density and node strength.	- The combined physical and cognitive training intervention led to significant changes in the density of the functional connectivity network in the beta frequency band for the LLM group, indicating neuroplastic changes in the brain. - The combined training led to a functional network with bilateral activations, particularly involving connections between frontal, occipital, and parietal regions, as well as some temporal regions. - The nodes of the functional network activated by the combined training were connected to brain regions associated with well-known resting state networks, which are known to be affected in Alzheimer’s disease.
Knoefel et al. (2018) [159]	- Patients with amnestic MCI, including those with amnestic MCI plus one additional affected cognitive domain - Mean education level of 15 years, which is higher than the Canadian average	To determine the feasibility of recruiting patients with MCI to test cognitive interventions	- 30 patients with amnestic MCI were planned to be divided into 2 intervention groups and 1 control group - Participants completed 1 h of brain training 3 times per week for 9 weeks - Outcome measures included recruitment, computer abilities, compliance, task performance, neuropsychological tests (RBANS, Trail Making, MoCA), and EEG/ERP - Neuropsychological and EEG/ERP assessments were conducted at baseline and post-intervention	- Recruitment of MCI participants for cognitive training interventions is challenging, but achievable. - Participants were able to learn and comply with the cognitive training interventions, despite some minor difficulties. - Participants showed improvements in their performance on the cognitive training tasks, but there were no significant changes on neuropsychological tests or EEG measures.
Lavy et al. (2021) [160]	- 30 participants total, 13 women and 17 men - Mean age of 71.93 years (SD = 8.51) - All participants diagnosed with MCI based on clinical and cognitive assessments - Inclusion criteria: age over 50 and MCI diagnosis - Exclusion criteria: active neurological pathology or axis one psychiatric disorder	- To use EEG-based neurofeedback to improve the memory performance of patients with MCI - To enhance the activity level of the upper alpha band through positive feedback	The study used a randomized controlled trial design with 30 participants diagnosed with MCI. Participants were randomly assigned to either an experimental group that received neurofeedback training to increase upper alpha power at the Pz electrode or a sham group that received random feedback from different electrodes. All participants underwent cognitive assessment using the NeuroTrax computerized battery before and after the 10 training sessions, as well as at a 30-day follow-up. EEG was recorded during the sessions using a 19-channel system.	- Participants in the neurofeedback training group showed a significant improvement in immediate verbal and non-verbal memory performance compared to the sham group, and this improvement was maintained for at least 1 month. - The improvement in memory performance was the only outcome measure that remained statistically significant after correcting for multiple comparisons. - There was no significant improvement in delayed verbal or non-verbal memory, or in other cognitive domains like executive function and attention.
Leite et al. (2022) [161]	- Community-dwelling adults aged 65 and older - Have cognitive complaints and decline in memory or other functions - Scored between 23 and 28 on the Mini-Mental State Examination - Do not have Alzheimer’s disease or other medical conditions that could impact cognitive decline or mortality - Do not have contraindications to tACS, severe sensory/communication difficulties, or prior cognitive training	- Assess if SoP training combined with α-tACS can increase speed of processing as measured by the UFOV, compared to SoP training or α-tACS alone - Assess if changes in speed of processing transfer to other cognitive domains like memory, language, and executive function using the NIH EXAMINER - Test the mechanisms underlying the interventions, specifically brain connectivity and coherence as measured by EEG	- Speed of processing (SoP) training plus active alpha-tACS - SoP training plus sham tACS - Active tACS alone Participants will be community-dwelling adults aged 65+ with cognitive complaints. They will receive 15 sessions over 6 weeks, with 9 sessions in the first 3 weeks and 2 sessions per week for the next 3 weeks. Outcomes will be assessed at baseline, week 3, week 6, and 1, 3, and 6 months after the intervention. The primary outcome is the Useful Field of View (UFOV) test, which measures speed of processing and attention. Secondary outcomes include the NIH EXAMINER battery to assess transfer effects to other cognitive domains, and EEG measures of brain connectivity and coherence.	
Makmee and Wongupparaj (2025) [162]	- Older adults, with a mean age of 66.31 ± 3.12 years for non-MCI older adults in the experimental group, 68.19 ± 5.03 years for older adults with MCI in the experimental group, and 64.97 ± 3.35 years for non-MCI older adults in the control group - Some participants had MCI, while others did not - The study involved an intervention aimed at enhancing cognitive functions and well-being in older adults	- Verbal and visuospatial short-term memory - Executive functions - Wellbeing in older adults with and without mild cognitive impairment	- An 8-session, 60 min per session, VR cognitive-based intervention held twice a week over 30 days - Participants included: ○ 31 non-MCI older adults in the experimental group (mean age 66.31 ± 3.12 years) ○ 29 older adults with MCI in the experimental group (mean age 68.19 ± 5.03 years) ○ 30 non-MCI older adults in the control group (mean age 64.97 ± 3.35 years) - Dependent variables were assessed using: ○ A battery of computerized tests ○ The well-being of older people questionnaire ○ Resting-state EEG	- The VR cognitive-based intervention led to significant improvements in short-term memory and executive functions in both MCI and non-MCI older adult groups. - The VR intervention enhanced well-being specifically in the older adults with MCI, but not in the non-MCI control group.
Marlats et al. (2020 [163]	- Older adults with MCI - At high risk of progressing to Alzheimer’s disease (AD) - 60 participants total, assigned to either an intervention or control group - Assessed using neuropsychological tests, questionnaires, and EEG at baseline, post-intervention, and 3-month follow-up	- Examine the effects of neurofeedback (NF) training on cognitive disorders, specifically targeting memory, attention functions, and brain electrical activity in elderly patients with MCI - Measure the primary outcome of change in attention using the Trail Making Test B - Measure secondary outcomes of changes in cognitive performance and EEG activities	- Randomized controlled trial (RCT) design - Single-blind protocol - Two groups: intervention group and control group - Intervention: 30 sessions of either sensorimotor/delta-ratio or beta1/theta-ratio neurofeedback training - Outcome measures: neuropsychological assessments, questionnaires, and EEG, measured at baseline, immediately after the intervention, and 3-month follow-up	
Marlats et al. (2019) [164]	- Elderly adults aged 65–90 years - Both men and women - Right-handed - At least 9 years of education - Diagnosed with MCI based on Petersen criteria - MMSE score > 20, indicating preserved global cognitive function - Performed below 1.5 SD of age/education norms on neuropsychological tests - Able to provide informed consent and had preserved daily living activities - No dementia or other major neurological/psychiatric disorders	- To assess whether neurofeedback (NF) training can improve cognitive performance in elderly patients with MCI - To investigate the effects of a SMR/theta NF training protocol on cognitive performance, psycho-affective scores, and EEG activity in elderly patients with MCI	- Recruited 33 right-handed patients aged 65–90 years with MCI from a memory center in Paris - Included patients with subjective memory complaints, MMSE > 20, MCI criteria, and preserved daily living activities - Excluded patients with neurological/psychiatric disorders, psychotropic medication, or participation in other trials - Conducted 20 neurofeedback training sessions over 10 weeks, 2 sessions per week, each lasting 1 h 15 min - Recorded EEG from the Cz electrode and trained participants to increase sensorimotor rhythm (SMR) while suppressing theta and beta waves - Analyzed changes in cognitive, psycho-affective, and EEG measures before, after, and 1 month after training	- Participants showed significant improvements in cognitive and psycho-affective measures, including memory, attention, and anxiety, after the neurofeedback (NF) training. - EEG recordings showed increased theta and alpha power after the NF training, which was sustained at the 1-month follow-up. - The study suggests that NF training can be effective in improving cognitive function and brain activity in elderly patients with mild cognitive impairment.
McNett et al. (2023) [165]	- Individuals with subjective cognitive complaints, MCI, or Alzheimer’s Disease (AD) - Some participants had other health issues that contributed to their discontinuation of the treatment - Varying degrees of cognitive impairment, ranging from subjective cognitive complaints to MCI and AD	- Examine the feasibility of 40 Hz sensory therapy delivered through a smart tablet application in a human population - Confirm the entrainment of 40 Hz stimulation in the cerebral cortex via EEG	- Participants used a smart tablet application to receive 40 Hz light and sound therapy for 1 h per day - EEG was used to confirm that the 40 Hz stimulation was detected in the participants’ cerebral cortex - 27 participants with subjective cognitive complaints, MCI, or Alzheimer’s disease were enrolled, and 11 completed the 6-month therapy - Participants’ cognitive function was assessed using the Montreal Cognitive Assessment (MOCA) and Boston Cognitive Assessment (BOCA)	- Difficulties with compliance and other health issues were common reasons for discontinuing the 40 Hz sensory therapy. - The therapy showed potential cognitive benefits for some patients with subjective cognitive complaints, MCI, and early Alzheimer’s disease, particularly in the domain of memory. - Subjective feedback from participants and caregivers indicated at least some perceived benefit from the therapy.
Mudar et al. (2019) [166]	- Older adults (age not specified) - Individuals with amnestic MCI	The study objectives were to examine the effects of Gist Reasoning training versus New Learning training on event-related neural oscillations (theta and alpha band power) during Go/NoGo tasks involving basic and superordinate semantic categorization in older adults with amnestic MCI.	- Between-groups design with Gist Reasoning training (*n* = 16) vs. New Learning training (*n* = 16) in older adults with amnestic MCI - Participants performed a Go/NoGo task with basic and superordinate semantic categorization - EEG was used to measure event-related neural oscillations (theta and alpha band power) before and after the training interventions - Both groups were compared at baseline and after training	- Both the Gist Reasoning training group and the New Learning training group showed increased event-related theta synchronization after their respective training programs. - The Gist Reasoning training group showed enhanced event-related desynchronization in low-frequency alpha band (8–10 Hz) on response inhibition (NoGo) trials and high-frequency alpha band (11–13 Hz) on response execution (Go) trials during superordinate categorization, relative to the New Learning group. - The Gist Reasoning training impacted neural processing related to strategic processing of the Go and NoGo trials during the more complex superordinate categorization task, in individuals with Mild Cognitive Impairment.
Oh et al. (2023) [167]	- 20 healthy young adults - 20 older adults with MCI	To determine the effectiveness of a multimodal brain empowerment (MBE) program in mitigating the modifiable risk factors in MCI To assess the therapeutic effects of the MBE program To evaluate the effects of the two multimodal interventions (TLC and REM) on healthy young adults and older adults with MCI	- Participants: 20 healthy young adults and 20 older adults with MCI - Intervention: Participants were randomly assigned to receive one of two multimodal brain empowerment (MBE) programs: tDCS, light therapy, and computerized cognitive therapy (TLC) Robot-assisted gait training, music therapy, and core exercise (REM) - Outcome measure: Electroencephalography (EEG) was used to assess changes in neural activity, including power spectrum and event-related synchronization (ERS) analysis, during the MBE interventions	- The multimodal brain empowerment (MBE) program, which included tDCS, light therapy, computerized cognitive therapy (TLC), and robot-assisted gait training, music therapy, and core exercise (REM), led to decreased delta waves and increased theta, alpha, and beta waves in the participants’ brain activity. - The MBE program resulted in increased neural activation in the frontal, temporal, and parietal lobes of the participants.
Rosales-Lagarde et al. (2018) [168]	- 6 older adults (OAs) with MCI, mean age 68.1 ± 3 - 7 control subjects without MCI, mean age 64.5 ± 9 - Similar in age and education level between MCI and control groups - Some participants had health conditions like hypertension, diabetes, and thyroid problems that were being treated	To explore the color of noise (fractal properties) using Detrended Fluctuation Analysis (DFA) and multichannel DFA (mDFA) in the transition from NREM to REM sleep in older adults with MCI compared to controls To compare the interhemispheric, anteroposterior, and posterior networks between the MCI and control groups during the NREM to REM transition To examine whether the stationarity of the EEG, EMG, and EOG signals could distinguish between the MCI and control groups	- Recruiting 6 older adults with MCI and 7 control (CTRL) older adults - Administering cognitive and emotional assessments, including the Neuropsi, MMSE, GDS, and SAST - Conducting one night of polysomnography on all participants to record EEG, EMG, and EOG data - Analyzing pre-REM and REM sleep epochs from the polysomnography data	- The MCI group showed increased color of noise (CN) in the electrooculogram (EOG) and frontotemporal areas of the electroencephalogram (EEG) during the transition from NREM to REM sleep compared to the control group, indicating a breakdown in the fractal structure of these signals in MCI. - The frontopolar interhemispheric and right anteroposterior networks also showed increased CN in the MCI group during this transition.
Steiner et al. (2018) [169]	- Community-dwelling older adults over the age of 60 years - Diagnosed with MCI due to Alzheimer’s disease - Total sample size of 80 participants, evenly divided between treatment and placebo groups	- Primary objective: Evaluate the efficacy of 12 weeks of treatment with Sailuotong (SLT) compared to placebo on cognition in older adults with MCI - Secondary objectives: ○ Assess the mechanisms of action of SLT via electroencephalography (EEG), autonomic function, brain blood flow, and inflammation ○ Assess the safety of SLT in older adults with MCI	- Randomized, double-blind, placebo-controlled trial - 80 participants recruited and randomly assigned 1:1 to treatment or placebo group - Primary cognitive outcome measures: Logical Memory Story A delayed recall, Letter Number Sequencing, Trail Making Test, Rey Complex Figure Test - Secondary outcome measures: EEG activity, autonomic function, brain blood flow, inflammatory cytokines	
Styliadis et al. (2015) [170]	- 70 seniors with MCI - Underwent neuropsychological screening prior to enrollment - Divided into 5 equally sized groups of 14 participants each - Matched across groups on age, years of education, gender ratio, and cognitive state (MMSE score)	- To investigate whether a combined cognitive and physical training can induce changes in cortical activity as measured by EEG - To determine if these EEG changes can index a deceleration of pathological brain aging processes in MCI patients - To test the hypothesis that the combined cognitive and physical training will show superior effects on EEG rhythms compared to single interventions	- Participants: 70 right-handed individuals with MCI, divided into 5 equally populated groups (14 participants per group) that underwent different training interventions - Interventions: The 5 groups underwent different training interventions, including combined cognitive and physical training, cognitive training only, physical training only, active control, and passive control - EEG data collection and analysis: A 5 min resting state EEG was recorded before and after the 8-week intervention, and cortical sources were modeled using exact low-resolution brain electromagnetic tomography (eLORETA) - Statistical analysis: Nonparametric statistical methods were used to compare the pre- and post-intervention EEG source activity within and between groups	- An 8-week combined physical and cognitive training program in MCI patients led to decreases in delta, theta, and beta brain rhythms in the precuneus/posterior cingulate cortex, which was associated with improvements in cognitive function. - The combined training was more effective than physical or cognitive training alone, suggesting that the physical activity component plays a key role in driving the neuroplastic changes. - The study provides evidence that even short-term combined physical and cognitive training has the potential to significantly improve daily life functioning in elderly individuals at risk for dementia.
Thapa et al. (2020) [171]	- Older adults aged 55–85 years, with a mean age of 72.5 ± 5.32 years - Diagnosed with MCI - 68 total participants, with 34 in the control group and 34 in the VR intervention group - Predominantly female (52 women, 16 men) - Stratified by age, gender, education level, and participating center to ensure balanced groups	- To investigate the effects of a virtual reality (VR) intervention program on cognitive function in older adults with MCI - To investigate the effects of a VR intervention program on brain function (as measured by resting-state EEG) in older adults with MCI - To investigate the effects of a VR intervention program on physical function (gait speed and mobility) in older adults with MCI	- Recruiting 68 participants with MCI and randomly assigning them to either a VR intervention group (*n* = 34) or a control group (*n* = 34), with randomization stratified by age, gender, education level, and participating center - The VR intervention group received 24 sessions of VR-based cognitive training over 8 weeks, with 3 sessions per week lasting 100 min each; the control group received an 8-session educational program on general health care - The VR training consisted of 4 game-based activities (juice making, crow shooting, fireworks, and love house) using an Oculus VR headset and hand controllers	- The VR game intervention improved cognitive and frontal brain function in older adults with MCI. - The VR intervention group showed significant improvements in executive function, as well as decreased theta power and theta/beta ratio in the brain, which are associated with reduced cognitive impairment and improved attention. - The VR intervention group also showed significant improvements in physical function, including gait speed and mobility, compared to the control group.
Trauberg et al. (2021) [172]	- Patients with Parkinson’s Disease and mild cognitive impairment (PD-MCI) - Sample size of 19 patients from one of the 4 centers in the multicenter study	- Examine the effect of cognitive training on executive functions in Parkinson’s Disease patients with mild cognitive impairment (PD-MCI) - Investigate resting-state EEG as a biomarker of the cognitive training effects - Explore the relationship between resting-state EEG activity and executive function performance, and the potential of using baseline EEG as a predictor of response to cognitive training	- 19 patients underwent resting-state EEG (128 channels) before and after a 6-week training session - EEG data from frontal, central, and temporal regions were analyzed for alpha and theta-delta activity, and how these related to a composite score of executive function over time - Data from all four centers of the larger multi-center study will be included in a source-based network analysis of the EEG	- The performance and improvement of executive functions in Parkinson’s disease patients with mild cognitive impairment correlated positively with high-frequency and negatively with low-frequency oscillations in the resting-state EEG. - Resting-state EEG can be used as a biomarker to track changes in neuropsychological performance, specifically executive function, over time in this patient population. - The authors plan to examine whether baseline resting-state EEG activity can predict the response to cognitive or movement training in Parkinson’s disease patients with mild cognitive impairment.
Trenado et al. (2023) [173]	- Parkinson’s disease patients with mild cognitive impairment (PD-MCI) - Sample size of 19 PD-MCI patients - 10 patients in the cognitive training group - 9 patients in the physical activity group	- To explore the joint effect of cognitive training (CT) and physical activity (PA) interventions on executive function (EF) and attention in Parkinson’s disease patients with mild cognitive impairment (PD-MCI) - To investigate the usefulness of resting state EEG as a neurophysiological biomarker of the joint effect of CT and PA on EF and attention in PD-MCI	- Participants: 19 Parkinson’s disease patients with mild cognitive impairment (PD-MCI), with 10 in a cognitive training (CT) group and 9 in a physical activity (PA) group - Data collection: Resting-state EEG and neuropsychological assessments of executive function (EF) and attention, collected before and after the interventions - EEG analysis: Focused on frontal cortical areas due to their relevance to cognitive function - Analyses: Examined the joint effect of the CT and PA interventions on EF and attention, as well as the relationships between EEG power in the theta and alpha bands and these cognitive measures	- A significant joint effect of cognitive training (CT) and physical activity (PA) interventions on executive function in Parkinson’s disease patients with mild cognitive impairment. - A trend towards a joint effect of CT and PA on attention in PD-MCI patients. - Resting state EEG measures of theta and alpha power in frontal areas can serve as a biomarker for the joint therapeutic effects of CT and PA interventions in PD-MCI patients.
Yang et al. (2022) [174]	- 99 participants with MCI - Mean age: 70.8 ± 5.4 years	The study objectives were to investigate the effectiveness of virtual-reality-based cognitive training (VRCT) and exercise on the brain, and the cognitive and physical activity of older adults with MCI. Specific outcomes measured included global cognitive function (MMSE), brain activity (resting-state EEG), and physical function (handgrip strength, gait speed).	- 99 participants with MCI were divided into 3 groups: VRCT, exercise, and control - The VRCT group received 24 sessions of virtual reality cognitive training, 3 days per week, with each session lasting 100 min - The exercise group received 24 sessions of aerobic and resistance training, 2 times per week, with each session lasting 60 min	- VRCT significantly improved global cognitive function as measured by the MMSE test in older adults with mild cognitive impairment. - Exercise training significantly improved both physical function (handgrip strength) and global cognitive function in older adults with mild cognitive impairment. - Exercise training led to a significant decrease in the theta/beta power ratio in the central brain region, indicating improved neural oscillatory activity, while VRCT also showed a decrease in this ratio, though not statistically significant.
Zhang et al. (2022) [175]	- Age: 55–75 years old - Handedness: Right-handed - Cognitive status: MCI meeting AAN diagnostic criteria, with MoCA scores between 18 and 26 - No severe organ dysfunction, psychiatric disorders, rTMS contraindications, dementia, or recent medication use affecting EEG	- To investigate the effect of dual-target rTMS (left DLPFC + left PCu) on improving cognition, particularly memory, in MCI patients - To assess the impact of dual-target rTMS on functional brain network connectivity in MCI patients using EEG	- Recruited 15 clinically diagnosed MCI patients and normal controls - Conducted resting-state EEG recordings and neuropsychological assessments before and after the intervention - Applied dual-target rTMS on the left dorsolateral prefrontal cortex (DLPFC) and left precuneus (PCu) with 10 Hz frequency, 1 s stimulation time and 10 s interval, 120 repetitions (1200 pulses) for DLPFC, and 80 repetitions (800 pulses) for PCu	- Dual-target rTMS increased the functional connectivity between the right posterior cingulate gyrus and the right dorsal caudate nucleus in MCI patients, which was associated with improvements in overall cognitive function, memory, executive function, and attention. - Functional connectivity between the right PCC and right DC was reduced in MCI patients compared to normal controls. - Dual-target rTMS significantly improved cognitive function in MCI patients.
Zhao et al. (2020) [176]	- Community-dwelling older adults, both men and women - Aged 60 years or older - Meet Petersen criteria for mild cognitive impairment - Exclude those with severe medical events during the study or who are unwilling to continue the training	Evaluate the effect of process-based multi-task cognitive training on executive function in older adults with MCI Explore the long-term effects and transfer effects of the cognitive training Explore the neural correlates underlying the changes in performance from the cognitive intervention	The study used a randomized controlled trial design with 90 participants with MCI randomly assigned to a cognitive training group or a wait-list control group. The cognitive training group received 10 weeks of process-based multi-task cognitive training and health education, while the control group received only health education. The primary outcome was executive function, with secondary outcomes of neuropsychological assessments and EEG measures, assessed at baseline, after 10 weeks, and 3-month follow-up.	
Ziloochi et al. (2024) [177]	- 26 individuals with MCI - Age: 63 ± 5 years - Gender: 4 females, 22 males - Randomly divided into a control group and a chiropractic intervention group, with 13 subjects in each group	- To investigate changes in neural activity using EEG signals before and after chiropractic or control intervention in participants with MCI - To use a randomized controlled cross-over design to compare the effects of chiropractic and control interventions	- Randomized controlled cross-over study design conducted at a hospital in Pakistan - Participants were individuals with MCI aged 63 ± 5 years, randomly divided into control and chiropractic intervention groups (*n* = 13 per group) - EEG data were recorded for 2 min at 2048 Hz from 62 channels, with participants instructed to focus on a fixation cross - Chiropractic spinal manipulation (SM) and control interventions were administered by an experienced chiropractor, similar to previous studies - EEG data were preprocessed to remove artifacts using independent component analysis and voltage/peak-to-peak voltage thresholds	- Chiropractic intervention led to a decrease in power in the delta and theta bands, and an increase in power in the beta2 band, which are signs of improvement in mild cognitive impairment. - The SVM classification method was able to distinguish the chiropractic and control groups with high accuracy, particularly in the beta2, theta, and delta bands. - Chiropractic intervention led to significant differences in interhemispheric coherence changes compared to the control group, suggesting it may have a positive effect on functional connectivity in the brains of MCI patients.

## Supplementary Materials

The following supporting information can be downloaded at: https://www.mdpi.com/article/10.3390/brainsci15060582/s1. Table S1. Experimental techniques across the RCTs (*n* = 34).

## Abbreviations

AD	Alzheimer’s Disease
AI	Artificial Intelligence
ANN	Artificial Neural Network
AR	Augmented Reality
BAN	Body Area Network
BCI	Brain–Computer Interface
CNN	Convolutional Neural Network
DLPFC	Dorsolateral Prefrontal Cortex
DL	Deep Learning
DNN	Deep Neural Network
DMN	Default Mode Network
ECG	Electrocardiography
EEG	Electroencephalography
EOG	Electrooculography
EMG	Electromyography
ERP	Event-Related Potential
ERSP	Event-Related Spectral Perturbation
ESCARF	Enhanced Simultaneous Cognitive-Physical Dual-Task Training Based on Fairy Tales
FCcANN	Fully Connected Cascade Artificial Neural Network
fMRI	Functional Magnetic Resonance Imaging
fNIRS	Functional Near-Infrared Spectroscopy
HGS	Hand Grip Strength
ICA	Independent Component Analysis
ITC	Inter-Trial Coherence
ITT	Intention-to-Treat
LLTM	Long Short-Term Memory
MCI	Mild Cognitive Impairment
ML	Machine Learning
MMSE	Mini-Mental State Examination
MoCA	Montreal Cognitive Assessment
MSC	Magnitude-Squared Coherence
NIBS	Non-Invasive Brain Stimulation
NF	Neurofeedback
NIRS	Near-Infrared Spectroscopy
NOS	Newcastle–Ottawa Scale
PCC	Posterior Cingulate Cortex
PCu	Precuneus
PLV	Phase Locking Value
PSG	Polysomnography
RBANS	Repeatable Battery for the Assessment of Neuropsychological Status
RoB	Risk of Bias
rs-fMRI	Resting-State Functional MRI
SDST	Symbol Digit Substitution Test
SCD	Subjective Cognitive Decline
SMR	Sensorimotor Rhythm
SoP	Speed of Processing
TES	Transcranial Electrical Stimulation
tACS	Transcranial Alternating Current Stimulation
TMT	Trail Making Test
tDCS	Transcranial Direct Current Stimulation
TMS	Transcranial Magnetic Stimulation
TR	Telerehabilitation
UFOV	Useful Field of View
VR	Virtual Reality
VRCT	Virtual Reality-Based Cognitive Training
VMR	Vasomotor Reactivity
WM	Working Memory
