# Supplementary material for: Neurotechnological Approaches to Cognitive Rehabilitation in Mild Cognitive Impairment: A Systematic Review of Neuromodulation, EEG, Virtual Reality, and Emerging AI Applications
Source: Brain Sci. 2025 May 28;15(6):582. doi: 10.3390/brainsci15060582 (PMC12190363; doi:10.3390/brainsci15060582)
Supplement: Supplementary file 1 [file brainsci-15-00582-s001.zip › Supplementary_Table_S1.pdf]

Supplementary Materials [S1]

Table S1. Experimental techniques across the RCTs (n=34)

Authors	Experimental techniques
Amjad et al. (2019a) [144]	- Randomized controlled trial design<br>- Experimental group played Xbox 360 Kinect cognitive games<br>- Control group performed range of motion exercises<br>- Assessments at 3 time points: before, after 1 session, and after 6 weeks<br>- Outcome measures:<br>- Mini-mental state examination (MMSE)<br>- Montreal cognitive assessment scale (MoCA)<br>- Trail making test (TMT) A and B<br>- Slowness and complexity of EEG
Amjad et al. (2019b) [145]	- EEG recording using the Emotiv EPOC® Headset with 14 channels and 2 references<br>- Mini-Mental State Examination (MMSE) and Montreal Cognitive Assessment (MoCA) neurocognitive tests<br>- Trail Making Test A and B (TMT-A and TMT-B) to assess executive functions
Babiloni et al. (2014) [146]	- Electroencephalography (EEG) to record resting-state brain activity<br>- Near-infrared spectroscopy (NIRS) to measure changes in cerebral blood oxygenation and haemodynamics<br>- Analysis of EEG coherence across multiple frequency bands (delta, theta, alpha 1, alpha 2, beta 1, beta 2, gamma)
Bae et al. (2024) [147]	- Two-channel prefrontal ERP recording using a portable device<br>- Auditory oddball task to elicit ERP signals<br>- ERP component analysis<br>- Connectivity analysis using phase locking value (PLV) and coherence (COH)<br>- Time-frequency analysis (ERSP and ITC)<br>- Time-trial analysis<br>- Grand average analysis<br>- Statistical analysis (t-tests, ANCOVA, post-hoc tests)
Cai et al. (2022) [148]	- Sleep quality measured by Pittsburgh Sleep Quality Inventory<br>- Cognitive function measured by Montreal Cognitive Assessment and Mini-Mental State Examination<br>- Assessment of secondary outcomes:<br>- Insomnia measured by Insomnia Severity Index<br>- Depression, anxiety, and perceived stress<br>- Collection and analysis of EEG data during mindfulness state
Caminiti et al. (2024) [149]	- 3D T1-weighted MRI<br>- 3D T2-weighted FLAIR MRI<br>- Resting-state functional MRI (rs-fMRI)<br>- High-density electroencephalography (HD-EEG)
Emonson et al. (2019) [150]	- Neuropsychological tasks<br>- Transcranial magnetic stimulation (TMS) combined with electroencephalography (EEG)<br>- Transcranial direct current stimulation (tDCS) applied to the left dorsolateral prefrontal cortex<br>- 2-Back working memory task with concurrent EEG recording
Han & Youn (2023) [151]	- Resting-state electroencephalography (EEG)<br>- Quantitative electroencephalography (qEEG)<br>- Comparison of qEEG data before and after administration of choline alphoscerate for 2 months
Hathaway et al. (2021) [152]	- Transcranial Electrical Stimulation (TES)<br>- Sham (placebo) stimulation<br>- All-night sleep EEG recordings<br>- Convolutional neural network<br>- EEG sleep staging<br>- Analysis of N3 sleep duration<br>- Analysis of spectral power in the 0.5-1 Hz frequency band

Hong et al. (2018) [153]	- Randomized controlled trial design with 4 groups: MCI exercise, MCI control, healthy exercise, and healthy control<br>- 12-week resistance exercise program using an elastic band at 15-repetition maximum (65% of 1RM)<br>- Measurement of electroencephalogram (EEG) patterns<br>- Neuropsychological tests<br>- Senior Fitness Test
Jiang et al. (2019) [154]	- Finger Tapping Test (FTT) and Purdue Pegboard Test (PPT) to measure psychomotor speed<br>- Montreal Cognitive Assessment (MoCA) to measure cognitive function<br>- Electroencephalography (EEG) to measure brain activity
Jung et al. (2022) [155]	- Electroencephalogram (EEG) to measure cognitive function<br>- Cerebral blood flow (CBF) to measure cognitive function<br>- O'Leary index to measure oral health<br>- Löe & Silness index to measure oral health<br>- Tongue coating assessment to measure oral health<br>- Unstimulated saliva flow rate to measure oral health<br>- Oral muscle strength assessment to measure oral health<br>- Mental health, happiness, and social support assessments to measure mental health status
Kim et al. (2023) [156]	- Korean version of the Montreal Cognitive Assessment<br>- Electroencephalography (EEG)<br>- Muscle strength<br>- Flexibility<br>- Agility<br>- Memory self-efficacy questionnaire<br>- Physical self-efficacy scale<br>- Quality of life
Kim et al. (2024) [157]	- Randomized controlled trial design<br>- Double-blinding (participants and researchers blinded to treatment conditions)<br>- Participants wore a dental mask with either phytoncide fragrance or water (control) for 30 minutes<br>- Quantitative electroencephalography (EEG) to measure resting-state brain activity before and after the intervention
Klados et al. (2016) [158]	- EEG recording with 57 electrodes placed according to the 10/10 international system<br>- REG-ICA method for removal of ocular artifacts<br>- Extended-ICA for decomposing EEG signals into independent components<br>- Boundary Element Model (BEM) for computing a generic head model<br>- Magnitude Square Coherence (MSC) for computing functional connectivity between cortical sources
Knoefel et al. (2018) [159]	- Cognitive training intervention, with participants completing brain training exercises for 1 hour, 3 times per week for 9 weeks<br>- Neuropsychological testing, including the Repeatable Battery for the Assessment of Neuropsychological Status (RBANS), Trail Making Test A & B, and Montreal Cognitive Assessment (MoCA)<br>- Electroencephalography (EEG) and event-related potentials (ERPs), with participants wearing EEG sensor caps and performing cognitive tasks like n-back, go-no-go, and verbal recognition paradigms
Lavy et al. (2021) [160]	- EEG-based neurofeedback to train participants to increase upper alpha band power at Pz<br>- Sham neurofeedback training as a control condition<br>- NeuroTrax TM computerized cognitive assessment battery<br>- Independent component analysis and visual examination to extract EEG artifacts<br>- 0.50-30 Hz rhythm pass filter applied to EEG data
Leite et al. (2022) [161]	- Pre-screening and clinical screening procedures to assess participant eligibility<br>- EEG to measure individual alpha frequency (IAF) and deliver alpha-tACS stimulation to the prefrontal cortex based on the IAF<br>- Resting-state EEG and an auditory oddball task to measure changes in brain activity and connectivity<br>- The NIH EXAMINER battery to assess transfer effects of the intervention to other

	cognitive domains<br>- The Useful Field of View (UFOV) test as the primary outcome measure for speed of processing and attention
Makmee & Wongupparaj (2025) [162]	- Virtual reality (VR) cognitive-based intervention delivered in 8 sessions over 30 days<br>- Experimental group with MCI participants, experimental group with non-MCI participants, and a control group of non-MCI participants<br>- Battery of computerized cognitive tests, well-being questionnaire, and resting-state EEG
Marlats et al. (2019) [163]	- Randomized controlled trial (RCT) design<br>- Two neurofeedback (NF) training protocols:<br>- Sensorimotor/delta-ratio NF training<br>- Beta1/theta-ratio NF training<br>- Psycho-pedagogical care as a control condition<br>- Neuropsychological assessments<br>- Questionnaires<br>- Electroencephalography (EEG) measurements<br>- Assessments at baseline, post-training, and 3-month follow-up
Marlats et al. (2020) [164]	- EEG recording from the Cz electrode location for SMR/theta neurofeedback training<br>- Use of an EEG Digitrack Biofeedback device to provide neurofeedback training<br>- 20 neurofeedback training sessions over 10 weeks, with each session lasting 1 hour 15 minutes<br>- Pre- and post-training EEG recording from 19 scalp locations using an electrocap<br>- Artifact removal using independent component analysis (ICA) and covariance-based approaches
McNett et al. (2023) [165]	- Use of a smart tablet application to deliver 40 Hz light and sound stimulation to participants for 1 hour per day<br>- EEG to confirm entrainment of the 40 Hz stimulation in the cerebral cortex<br>- Montreal Cognitive Assessment (MOCA) and Boston Cognitive Assessment (BOCA) to evaluate participants' cognitive function
Mudar et al. (2019) [166]	- Electroencephalography (EEG) to measure neural oscillations in the theta and alpha frequency bands<br>- Go/NoGo task to assess response inhibition and execution<br>- Pre-post design to evaluate changes in neural oscillations before and after training<br>- Comparison of two training interventions: Gist Reasoning training and New Learning training
Oh et al. (2023) [167]	- EEG power spectrum analysis<br>- EEG event-related synchronization (ERS) analysis<br>- tDCS<br>- Light therapy<br>- Computerized cognitive therapy (TLC)<br>- Robot-assisted gait training<br>- Music therapy<br>- Core exercise
Rosales-Lagarde et al. (2018) [168]	- Electroencephalography (EEG) using 19 electrodes placed according to the International 10-20 System<br>- Electromyography (EMG) using 2 electrodes on the chin<br>- Electrooculography (EOG) using 2 electrodes around the eyes<br>- A leg electrode to detect Restless Leg Syndrome<br>- Filtering of the signals (0.1-100 Hz for EEG, 10-70 Hz for EMG, 0.3-15 Hz for EOG, with a 60 Hz notch filter)<br>- Digitization of the signals at 512 Hz with 16-bit resolution<br>- Data analysis using MATLAB, including Detrended Fluctuation Analysis (DFA) and multichannel DFA (mDFA)
Steiner et al. (2018) [169]	- Electroencephalography (EEG)<br>- Electrocardiogram<br>- Skin conductance<br>- Peripheral pulse pressure<br>- Common carotid artery ultrasound<br>- Serum cytokine measurements<br>- Logical Memory Story A delayed recall

	- Letter Number Sequencing<br>- Trail Making Test<br>- Rey Complex Figure Test
Styliadis et al. (2015) [170]	- Resting-state EEG recording<br>- eLORETA source modeling of EEG data<br>- ICA for artifact removal<br>- SnPM for statistical analysis of eLORETA source data<br>- Spearman's correlation to relate MMSE changes to eLORETA source changes
Thapa et al. (2020) [171]	- Mini-Mental State Examination (MMSE)<br>- Trail Making Test (TMT) A & B<br>- Symbol Digit Substitution Test (SDST)<br>- Resting-state electroencephalogram (EEG) with a 19-channel wireless device<br>- Virtual reality (VR) training using an Oculus VR headset and hand controllers<br>- Gait speed test<br>- 8-feet Up and Go test for mobility<br>- Handgrip strength measurement using a digital dynamometer
Trauberg et al. (2021) [172]	- Resting-state EEG with 128 channels<br>- Analysis of frontal, central, and temporal EEG activity in the alpha and theta-delta frequency bands<br>- Relating EEG measures to a composite score of executive function performance<br>- Planned source-based network analysis of EEG data from all four centers in the larger multi-center study
Trenado et al. (2023) [173]	- Resting state EEG<br>- Neuropsychological assessments of executive function (EF) and attention<br>- EEG analysis focused on frontal cortical areas<br>- Examination of the relationship between EEG measures (theta and alpha power) and neuropsychological outcomes (EF and attention)
Yang et al. (2022) [174]	- Virtual-reality-based cognitive training (VRCT)<br>- Exercise intervention (aerobic and resistance training)<br>- Mini-Mental State Examination (MMSE) test for cognitive function<br>- Resting-state electroencephalography (EEG) for neurophysiological assessment<br>- Handgrip strength (HGS) and gait speed for physical function assessment
Zhang et al. (2022) [175]	- Repetitive transcranial magnetic stimulation (rTMS) targeting the left dorsolateral prefrontal cortex (DLPFC) and the precuneus (PCu)<br>- Resting-state EEG recordings to measure brain activity<br>- Source-level EEG data analysis using the DICS algorithm and the Brainnetome atlas<br>- Functional connectivity analysis using phase locking value (PLV)
Zhao et al. (2020) [176]	- Participant recruitment through community outreach and advertisements<br>- Screening assessments including cognitive tests (MMSE, MoCA)<br>- Physician-administered clinical assessments to diagnose MCI<br>- Computer-generated randomization to assign participants to intervention and control groups
Ziloochi et al. (2024) [177]	- EEG recording from 62 channels at 2048 Hz sampling rate<br>- Chiropractic spinal manipulation (SM) intervention<br>- Sham/control intervention involving passive movements without manipulation
